# *Lansium domesticum*—A Fruit with Multi-Benefits: Traditional Uses, Phytochemicals, Nutritional Value, and Bioactivities

**DOI:** 10.3390/nu14071531

**Published:** 2022-04-06

**Authors:** Hossam M. Abdallah, Gamal A. Mohamed, Sabrin R. M. Ibrahim

**Affiliations:** 1 Department of Natural Products and Alternative Medicine, Faculty of Pharmacy, King Abdulaziz University, Jeddah 21589, Saudi Arabia; hmafifi@kau.edu.sa; 2 Department of Chemistry, Preparatory Year Program, Batterjee Medical College, Jeddah 21442, Saudi Arabia; sabrin.ibrahim@bmc.edu.sa; 3 Department of Pharmacognosy, Faculty of Pharmacy, Assiut University, Assiut 71526, Egypt

**Keywords:** *Lansium domesticum*, Meliaceae, traditional uses, nutritional value, phytoconstituents, bioactivities

## Abstract

*Lansium domesticum* (Langsat, Meliaceae) is a tropical fruit mainly found in Southeast Asian countries, particularly in Thailand, Malaysia, Indonesia, and the Philippines. Traditionally, it is utilized as a folk treatment for eye inflammation, ulcers, diarrhea, dysentery, fever, spasms, flatulence, worms, insect bites, scorpion stings, and malaria. Additionally, it is utilized as a mosquito repellent, skin moisturizer and whitening agent. Pharmacological research showed that the plant has a wide array of bioactivities, including antimalarial, antifeedant, anti-aging, wound healing, antioxidant, cytotoxic, analgesic, antibacterial, antimutagenic, insecticidal, and larvicidal. The most commonly described activities were attributed to the presence of terpenoids and phenolics. Further, some studies reported the preparation of nanoparticles and pharmaceutical formulations from the plant. This review highlights the potential of *L. domesticum* as herbal medicine. It provides an overview about the reported data on *L. domesticum* from 1931 to November 2021, including nutritional value, traditional uses, phytoconstituents, and bioactivities, as well as nanoparticles and pharmaceutical formulations.

## 1. Introduction

Fruits, vegetables, and medicinal herbs are the richest sources of health-promoting compounds such as vitamins, β-carotene, minerals, flavonoids, phenolics, and polyphenolics that exert significant bioactivities [1,2]. Genus *Lansium* belongs to the Meliaceae family, which includes about 560 species and 50 genera that are widespread in tropical and subtropical regions [3]. Genus *Lansium* commonly recognized species are *Lansium breviracemosum* Kosterm., *L. membranaceum* (Kosterm.) Mabb., and *L. domesticum* Corrêa. [4]. This genus is represented by only one species, *L. domesticum*, in Peninsular Malaysia [4]. While in Java, it is represented by two species; *L. domesticum* Corrêa and *L. humile* Hassk., as well as a variety *L. domesticum* var. *pubescens* Koorders et Valeton have been recognized [5,6]. *L. domesticum* is a common evergreen Southeast Asian tree that occurs both in the wild or cultivated in these regions, where it represents one of the commonly cultivated fruits [7]. It has high market potential and adequate economic value in Southeast Asian countries. Thailand, Malaysia, Indonesia, and the Philippines are considered to be the main producers of *L. domesticum*. Additionally, the plant is cultivated in Burma, Vietnam, Puerto Rico, Sri Lanka, India, Hawaii, Surinam, and Australia [5,8,9]. *L. domesticum* Correa is a complicated aggregate species of different plant forms. It’s four prevalent types are Duku, Dokong (longkong), Duku-langsat, and Langsat. Duku and Langsat are the two most common types. Duku-langsat, Langsat, and Duku are domestic to Peninsular Malaysia, however, Dokong is found in southern Thailand and has been cultured in Peninsular Malaysia for >10 years [5,7]. The Duku-langsat is an intermediate type, it is conventionally regarded as uppermost type to both Duku and Langsat [4,7]. *L. domesticum* includes two botanically distinct varieties; var. *pubescens* and var. *domesticum* (Table 1) [10].

The plant has different synonyms; *Aglaia domestica* (Correa) Pellegrin, *A. aquea* (Jack) Kosterm., *A. intricatoreticulata* Kosterm., *A. dookoo* Griff., *A. merrillii* Elmer, *A. steenisii* Kosterm., *A. sepalina* (Kosterm.) Kosterm., *Lachanodendron domesticum* Nees, *Lansium domesticum* var. *aqueum* Jack, *L. aqueum* (Jack) M.Roem., *L. domesticum* var. *typicum* Backer, *L. domesticum* var. *pubescens* Koord. *& Valet.*, *L. javanicum* M. Roem., *L. javanicum* Koord. & Valet. ex Moll & Janss., *L. parasiticum* var. *aqueum* (Jack) Sahni & Bennet, *L. sepalinum* Kosterm, *L. parasiticum* Sahni & Bennet, *L. pedicellatum* Kosterm., and *Taeniochlaena polyneura* Schellenberg. Additionally, different local names have been given for *L. domesticum* [5,15,16] (Table 2).

Its tree has a 40–50 ft height with long leaves which are dark green and pinnate with a glossy surface. The flowers are present in clusters on the old branches and trunk of the tree. They are mostly bisexual, small with a yellow-white color. The fruits grow in clusters and are small, round (3–5 cm diameter) with a leathery yellow skin that can be thin or thick. The fruit’s flesh is translucent and juicy with six or five segments which have seeds. The fruits may be sweet or acidic relying on the growing conditions and variety [5]. The delicious, succulent, fruit aril is eaten fresh directly after peeling or can also be candied or preserved in syrup [5,17,18]. The jams, juices, sherbet, and ice creams are the most popular langsat products. On the contrary, the seeds and peel are the main byproducts after the flesh’s consumption, neither of which are widely used. However, the seeds and peels are a rich pool of bio-metabolites [12]. In Indonesia, the fruit is a very popular dessert, and the peel was traditionally known to be toxic to domestic animals [19]. The plant extracts exhibited various biological activities, including antimalarial, antifeedant, anti-aging, wound healing antioxidant, cytotoxic, analgesic, antibacterial, antimutagenic, insecticidal, and larvicidal. Phytochemical studies of *L. domesticum* indicated that triterpenoids particularly onoceranoids with unusual and unrivaled skeleton, cycloartenoid, and tetranortriterpenoid are the main constituents reported from this plant that displayed remarkable bioactivities.

In recent decades, herbal medicines have substantiated their publicity among consumers for both traditional and cultural reasons. Herbal medicines have been utilized for treating various ailments and diseases in many populations for thousands of years. They are considered the main treatment approach in many countries because of their safety, reliability, and affordability in comparison to synthetic ones that can cause adverse effects on human health. *L. domesticum* has immense role in providing medicinal and realistic value in many developing countries particularly in regions where medicine is unreachable, and the populations are in the need of healthcare. Thus, this review is aimed at describing and summarizing the studies on *L. domesticum*, including traditional uses, nutritional value, phytoconstituents, and bioactivities, as well as the production and season and nanoparticles and pharmaceutical formulations. The cited literature in the current work is dated from 1931 to November 2021.

## 2. Research Methodology

The reported data about *L. domesticum* was obtained through searching in various databases, including Web of Science, PubMed, Scopus, and Google scholar. Moreover, published papers in different publishers such as ACS, Elsevier, Bentham, Sage, Wiley, Taylor &Francis, Thieme Medical, and Springer were surveyed. Further, non-English papers, theses, conferences, and symposiums have been reviewed. The used keywords include *L. domesticum*, traditional uses, phytochemistry, bioactive compounds, pharmacology, and other related words. All of the conducted research from 1931 to 2021 has been reviewed.

## 3. Production and Season

Generally, *L. domesticum* produces fruit once and sometimes twice a year with differences in fruiting periods according to the area. In Malaya, it bears fruits twice a year, in June and July and again in December and January or even till February. In Indonesia, the plant is available anywhere during the rainy season (January to April) [19]. In the Philippines, the season is short and most of the fruits are off the market in >1 month, however, in India, the fruits become mature between April and September [20]. Its harvest season in Thailand is commonly between August and September of each year. Its production often varies from year to year, relying on the existence of a dry period for inducing flowering. The average production is only 1 ton/rai/year in Thailand. In Indonesia, the production reached 228,817 tons which placed it in the twelfth position of fruit production. In Southern Sumatra, the production reached 8419.1 tons in 2011 [21]. On the other hand, the average production was 1000 fruits/tree/year in the Philippines and an average 13.5 kg/trees produced annually in Nilgiris, India [20].

## 4. Traditional Uses of *L. domesticum*

The different parts of *L. domesticum* have various medicinal and non-medicinal uses in many nationalities (Table 3). The peel is wealthy in non-toxic oleoresin that is utilized against diarrhea and fevers [8]. In Thailand, the peel and flesh have been used as facial toners, wash gels, and masks, as well as a skin moisturizer and whitening cream. Additionally, the seeds possess antifeedant and febrifugal capacities and pericarp is utilized for repelling mosquitoes [22,23]. *L. domesticum* bark was used by people in the Pakuli region of Palu for malaria treatment. Moreover, the boiled bark with water was utilized to reduce pain and fever [24].

## 5. Nutritional Value of *L. domesticum*

The fruit tastes sweet and sour. It has a sour taste due to its low pH at about 3.85 that is aligns with the reported total acidity of fruit ≈1.04% [36]. Its taste has been resembled to a combination of grapefruit and grape and is considered excellent by most people. Its fructose, sucrose, and glucose contents are accountable for the sweet taste [37]. The fruit is a prosperous source of minerals, fats, protein, organic acids, carbohydrates, fiber, and vitamins. Various studies reported the evaluation of the nutritional value of this fruit. Chemical composition and mineral contents of flesh, peel, and seed of a fruit sample collected from Kuala Terengganu, Malaysia using ICP-OES (inductively couple plasma optical emission spectrometry) were previously evaluated [38]. The seeds had the highest crude protein (3.0 g/100 g), carbohydrates, and sodium, whereas the peels possessed high contents of crude fat, ash, calcium, potassium, and magnesium [38]. Furthermore, the seeds are rich in starch. Additionally, it was reported that the seeds and peels could have higher nutrient contents than pulp fruits [39]. In Thailand, the nutrient composition per100 g langsat fruit had energy (66 cal), moisture (82.9%), protein (0.9 g), fat (0.1 g), fibre (0.3 g), carbohydrate (15.3 g), Ca (5 mg), Fe (0.7 mg), P (35 mg), vitamin A (15 I.U.), vitamin B2 (0.02 mg), vitamin B1 (0.08 mg), niacin (0.1 mg), and vitamin C (46 mg) [40]. In addition, it was found that 100 g edible portion of duku showed 34 kcal energy, 90 g water, 0.4 g protein, 0.0 g fat, 8.2 g carbohydrate, 0.9 g fiber, 0.5 g ash, 10 mg Ca, 20 mg P, 1.0 mg Fe, 12 mg Na, 230 mg K, 0.05 mg vitamin B1, 0.02 mg vitamin B2, 0.5 mg niacin, and 13.4 mg vitamin C [41]. Meanwhile, 100 g longkong fruit flesh contained protein 1.0 g and crude fat 0.5 g, which are higher than that of duku and langsat fruit [18,42]. Moreover, 100 g of longkong contains water 84 g, fiber 0.8 g, carbohydrates 14.2 g, Ca 19 mg, ash 0.6 g, K 275 mg, and vitamins (B2, B1, and C). The energy value is 238 kJ/100 g [16,43,44]. It is noteworthy that sodium, magnesium, potassium, zinc, calcium, iron, and manganese are the major minerals in the fruit [12,45].

## 6. *L. domesticum* Enzymes

Enzymes are important biocatalysts in food biotechnology. Plant-derived enzymes (e.g., bromelain, invertase, amylase, papain, ficin, lipoxygenase, etc.) have played a remarkable role in various food industries, for example, dairy and bakery products, syrups, and alcoholic beverages. Besides, the plants can also be used as raw materials for enhancing the potential of the microbial enzyme that are employed in the food industry. *L. domesticum* fruit and pericarp are wealthy, with different active enzymes. On the other hand, these enzymes could contribute to the spoilage of the fruit. The fruits activated these enzymes for protection when they suffer from changes in the environment and/or storage temperature [12]. For example, oxidoreductases are activated when the peel or fruit is damaged. Phenylalanine ammonia-lyase, polyphenol oxidase, and peroxidase that are found in the pericarp oxidize the phenols to yield browning compounds [46,47]. Chitinase and β-1,3-glucanase are reported from the fruit peel that possessed antifungal potential towards *Metarhizium guizhouense* [48]. Polygalacturonase (PG) and pectin methylesterase (PME), as well as antioxidant enzymes: GPX (glutathione peroxidase), SOD (superoxide dismutase), and catalase (CAT) were detected in fully matured fruit that possessed high activities during fruit maturation [49]. Furthermore, the fruit had LOX (lipoxygenase) that is accountable for the polyunsaturated fatty acids deoxygenation and converting them into fragrance and signaling molecules for regulating leukotriene [50]. It was reported that polygalacturonase, pectin methylesterase, and cellulases rise the sugar profile in the fruit and decrease the firmness of the fruit during ripening [49].

## 7. Phytoconstituents of *L. domesticum*

The chemical investigation of various parts of *L. domesticum* resulted in the isolation of different chemical constituents; most of them have been isolated from the peels, seeds, and barks (Table 4). Their identification was carried out using various spectroscopic techniques, as well as X-ray and chemical means. A total of 112 compounds have been reported from *L. domesticum* (excluding nutrients such as amino acids, protein, and sugars), including various classes of triterpenoids (e.g., swietenine, onoceranoid, cycloatanoid, and tetranortriterpenoid), cardenolides, steroids, sesquiterpenes, organic acids, phenolics, and volatile compounds. It was reported that the fruit peel had an abundant level of reductive substances, glycosides, organic acids, alkaloids, flavonoids, and phenolics, but it had no saponins [51,52]. Phytochemical screening of the bark revealed the existence of anthraquinones, alkaloids, flavonoids, coumarins, cardiac glycosides, tannins, saponins, and iridoids [24]. Further, a toxic constituent such as lansium acid (6%) was detected in the peel [52,53]

### 7.1. Volatile Organic Compounds and Organic Acids

Volatile organic compounds (VOCs) are the small molecular weight lipophilic molecules with a low boiling point and volatility which result from the plant’s secondary and primary metabolism [54]. They include alcohols, terpenes, alkanes, olefins, aldehydes, and fatty acid derivatives [55].

The volatile constituents of langsat and duku fruits were obtained using vacuum distillation with subsequent extraction of the distillates by CH_2_Cl_2_ were analyzed by capillary GC and GC-MS. The results revealed that sesquiterpenes represented the dominant chemical class of volatiles in both langsat and duku fruits (77.14 and 89.21%, respectively) of which germacrene D (**1**) was the most abundant component [11]. Headspace-solid phase microextraction with the GCMS analysis of the juice from fruit obtained from Eastern Thailand revealed the presence of 43 volatiles among them 3-carene (**2**), δ-selinene (**3**), 1,3,5 trioxane (**4**), (E)-2-hexenal (**5**), α-cubebene (**6**), isoledene (**7**), and α-calacorene (**8**) were the major volatiles [56]. Longkong‘s fresh peel contains 0.2% of the brown resin, light-yellow volatile oil, and reducing acids. Whilst the dried peel contains semiliquid dark oleoresin composed of 22% resin and 0.17% volatile oil [16,44].

**Table 4:** List of the reported phytoconstituents from *Lansium domesticum*.

Compound Name	Chemical Class	Plant Part	Extract/ Fraction	Mol. Wt.	Mol. Formula	City, Country	Ref.
Germacrene D (**1**)	Sesquiterpene	Fruits	Essential oil	204	C_15_H_24_	Seepoa village, Narathiwat, Thailand	[57]
		Fruits	Essential oil	-	-	Penang, Malaysia	[11]
		Seeds	CH_2_Cl_2_/acetone	204	C_15_H_24_	Laguna, Philippine	[58]
3-Carene (**2**)	Monterpene	Fruits	Juice	136	C_10_H_16_	Eastern Thailand	[56]
δ-Selinene (**3**)	Sesquiterpene	Fruits	Juice	204	C_15_H_24_	Eastern Thailand	[56]
1,3,5-Trioxane (**4**)	Organic compound	Fruits	Juice	90	C_3_H_6_O_3_	Eastern Thailand	[56]
(*E*)-2-Hexenal (**5**)	Aldehyde	Fruits	Juice	98	C_6_H_10_O	Eastern Thailand	[56]
*α*-Cubebene (**6**)	Sesquiterpene	Young fruit	CHCl_3_	204	C_15_H_24_	Narathiwat, Satun, and Yala, Thailand	[43]
		Fruits	Juice	-	-	Eastern Thailand	[56]
Isoledene (**7**)	Sesquiterpene	Fruits	Juice	204	C_15_H_24_	Eastern Thailand	[56]
*α*-Calacorene (**8**)	Sesquiterpene	Fruits	Juice	200	C_15_H_20_	Eastern Thailand	[56]
Ethyl oleate (**9**)	Fatty acid ester	Young fruit	CHCl_3_	310	C_20_H_38_O_2_	Narathiwat, Satun, and Yala, Thailand	[43]
Hexadecenoic acid (**10**)	Fatty acid	Young fruit	CHCl_3_	254	C_16_H_30_O_2_	Narathiwat, Satun, and Yala, Thailand	[43]
1,2,4a,5,6,8a-Hexahydro-4,7-dimethyl-1-(1-methylethyl)-naphthalene (**11**)	Sesquiterpene	Young fruit	CHCl_3_	204	C_15_H_24_	Narathiwat, Satun, and Yala, Thailand	[43]
Octadecanoic acid (**12**)	Fatty acid	Young fruit	CHCl_3_	284	C_18_H_36_O_2_	Narathiwat, Satun, and Yala, Thailand	[43]
α-Copaene (**13**)	Sesquiterpene	Fruits	Essential oil	204	C_15_H_24_	Seepoa village, Narathiwat, Thailand	[57]
Oleic acid (**14**)	Fatty acid	Fruits	MeOH	282	C_18_H_34_O_2_	Seepoa village, Narathiwat, Thailand	[57]
δ-Cadinene (**15**)	Sesquiterpene	Fruits	Essential oil	204	C_15_H_24_	Seepoa village, Narathiwat, Thailand	[57]
τ-Muurolol (**16**)	Sesquiterpene	Fruits	Essential oil	222	C_15_H_26_O	Seepoa village, Narathiwat, Thailand	[57]
Palmitic acid (**17**)	Fatty acid	Fruits	MeOH	256	C_16_H_32_O_2_	Seepoa village, Narathiwat, Thailand	[57]
(+)-Spathulenol (**18**)	Sesquiterpene	Fruits	Essential oil	220	C_15_H_24_O	Seepoa village, Narathiwat, Thailand	[57]
Citric acid (**19**)	Organic acid	Fruits	H_2_O	192	C_6_H_8_O_7_	North Sulawesi, Indonesia	[36]
Malic acid (**20**)	Organic acid	Fruits	H_2_O	134	C_4_H_6_O_5_	North Sulawesi, Indonesia	[36]
Piroglutamic acid (**21**)	Organic acid	Fruits	H_2_O	129	C_5_H_7_NO_3_	North Sulawesi, Indonesia	[36]
Ascorbic acid (**22**)	Organic acid	Fruits	H_2_O	176	C_6_H_8_O_6_	North Sulawesi, Indonesia	[36]
Glycolic acid (**23**)	Organic acid	Fruits	MeOH	76	C_2_H_4_O_3_	Seepoa village, Narathiwat province, Thailand	[57]
Maleic acid (**24**)	Organic acid	Fruits	MeOH	116	C_4_H_4_O_4_	Seepoa village, Narathiwat province, Thailand	[57]
Ferulic acid (**25**)	Phenolic acid	Fruits	MeOH	194	C_10_H_10_O_4_	Singapore	[59]
*P*-Coumaric acid (**26**)	Phenolic acid	Fruits	MeOH	164	C_9_H_8_O_3_	Singapore	[59]
Gallic acid (**27**)	Phenolic acid	Fruits	MeOH	170	C_7_H_6_O_5_	Singapore	[59]
Ellagic acid (**28**)	Phenolic acid	Fruits	MeOH	302	C_14_H_6_O_8_	Singapore	[20]
Chlorogenic acid (**29**)	Phenolic acid	Fruit peels	LDSK50-EA LDSK50-H_2_O	354	C_16_H_18_O_9_	Prathumthani, Thailand	[51]
Rutin (**30**)	Flavonoid	Fruit peels	LDSK50-EA LDSK50-H_2_O	610	C_27_H_30_O_16_	Prathumthani, Thailand	[51]
Scopoletin (**31**)	Coumarin	Fruit peels	LDSK50-EA LDSK50-H_2_O	192	C_10_H_8_O_4_	Prathumthani, Thailand	[51]
Quercetin (**32**)	Flavonoid	Fruit peels	LDSK50-EA LDSK50-H_2_O	302	C_15_H_10_O_7_	Prathumthani, Thailand	[51]
Catechin (**33**)	Flavonoid	Fruit peels	LDSK50-EA LDSK50-H_2_O	290	C_15_H_14_O_6_	Prathumthani, Thailand	[51]
Lansic acid (**34**)	Onoceranoid triterpenoid	Fruit peels	EtOH/CH_2_Cl_2_	470	C_30_H_46_O_4_	Bogor, Indonesis	[19,60]
	Onoceranoid triterpenoid	Fruit peels	MeOH/EtOAc	-	-	Khon Kaen, Thailand	[61]
	Onoceranoid triterpenoid	Leaves	MeOH/EtOAc	-	-	Ba’kelalan, Sarawak, Malaysia	[62]
Lansioside A (**35**)	Onoceranoid triterpenoid	Fruit peels	EtOH/CH_2_Cl_2_	659	C_38_H_61_NO_8_	Bogor, Indonesia	[19,60,63]
	Onoceranoid triterpenoid	Leaves	MeOH/EtOAc	-	-	Ba’kelalan, Sarawak, Malaysia	[62]
	Onoceranoid triterpenoid	Fruit peels	EtOH	-	-	Japan	[64]
Lansioside B (**36**)	Onoceranoid triterpenoid	Fruit peels	EtOH/CH_2_Cl_2_	618	C_36_H_58_O_8_	Bogor, Indonesia	[19,60]
	Onoceranoid triterpenoid	Fruit peels	EtOH	-	-	Japan	[64]
	Onoceranoid triterpenoid	Seeds	CH_2_Cl_2_/EtOAc	-	-	Thumbon Nopitum, Nakhon Si Thammarat, Thailand	[65]
	Onoceranoid triterpenoid	Leaves	MeOH/EtOAc	-	-	Ba’kelalan, Sarawak, Malaysia	[62]
Lansioside C (**37**)	Onoceranoid triterpenoid	Fruit peels	EtOH/CH_2_Cl_2_	588	C_35_H_56_O_7_	Bogor, Indonesia	[19,60]
	Onoceranoid triterpenoid	Fruit peels	EtOH	-	-	Japan	[64]
	Onoceranoid triterpenoid	Fruit peels	CH_2_Cl_2_/EtOAc	-	-	Laguna, Philippine	[58]
	Onoceranoid triterpenoid	Leaves	MeOH/EtOAc	-	-	Ba’kelalan, Sarawak, Malaysia	[62]
Methyl lansiolate (**38**)	Onoceranoid triterpenoid	Fruit peels	EtOH/CH_2_Cl_2_	470	C_31_H_50_O_3_	Bogor, Indonesia	[19]
	Onoceranoid triterpenoid	Fruit peels	MeOH/EtOAc	-	-	Ra-ngae, Narathiwat, Thailand	[66]
	Onoceranoid triterpenoid	Leaves	MeOH/EtOAc	-	-	Ba’kelalan, Sarawak, Malaysia	[62]
Lansiolic acid (**39**)	Onoceranoid triterpenoid	Fruit peels	EtOH/CH_2_Cl_2_	456	C_30_H_43_O_3_	Bogor, Indonesia	[19]
	Onoceranoid triterpenoid	Leaves	MeOH/EtOAc	454	C_30_H_46_O_3_	Indonesia	[67]
	Onoceranoid triterpenoid	Fruit peels and seeds	CH_2_Cl_2_/EtOAc	-	-	Laguna, Philippine	[58]
	Onoceranoid triterpenoid	Seeds	CH_2_Cl_2_/EtOAc	-	-	Thumbon Nopitum, Nakhon Si Thammarat, Thailand	[65]
	Onoceranoid triterpenoid	Bark	EtOH/EtOAc	-	-	Apo Kayan Indonesia	[68]
	Onoceranoid triterpenoid	Fruit peels	MeOH/EtOAc	-	-	Ra-ngae, Narathiwat, Thailand	[66]
	Onoceranoid triterpenoid	Leaves	MeOH/EtOAc	-	-	Ba’kelalan, Sarawak, Malaysia	[62]
Dukunolide A (**40**)	Tetranortriterpenoid	Seeds	CH_2_Cl_2_/*n*-Hexane (Duku)	482	C_26_H_26_O_9_	Bogor, Indonesia	[69,70]
		Seeds	MeOH/EtOAc	-	-	Pontianak, West Kalimantan, Indonesia	[71]
Dukunolide B (**41**)	Tetranortriterpenoid	Seeds	CH_2_Cl_2_/*n*-Hexane	498	C_26_H_26_O_10_	Bogor, Indonesia	[70]
		Seeds	CH_2_Cl_2_/EtOAc	-	-	Thumbon Nopitum, Nakhon Si Thammarat, Thailand	[65]
		Seeds	MeOH/EtOAc	-	-	Pontianak, West Kalimantan, Indonesia	[71]
Dukunolide C (**42**)	Tetranortriterpenoid	Seeds	CH_2_Cl_2_/*n*-Hexane	540	C_28_H_28_O_11_	Bogor, Indonesia	[70]
		Seeds	CH_2_Cl_2_/EtOAc	-	-	Thumbon Nopitum, Nakhon Si Thammarat, Thailand	[65]
		Seeds	MeOH/EtOAc	-	-	Pontianak, West Kalimantan, Indonesia	[71]
Dukunolide D (**43**)	Tetranortriterpenoid	Seeds	CH_2_Cl_2_/*n*-Hexane	468	C_26_H_28_O_8_	Bogor, Indonesia	[72]
		Seeds	CH_2_Cl_2_/EtOAc	-	-	Thumbon Nopitum, Nakhon Si Thammarat, Thailand	[65]
		Seeds	MeOH/EtOAc	-	-	Pontianak, West Kalimantan, Indonesia	[71]
Dukunolide E (**44**)	Tetranortriterpenoid	Seeds	CH_2_Cl_2_/*n*-Hexane	484	C_26_H_28_O_9_	Bogor, Indonesia	[72]
Dukunolide F (**45**)	Tetranortriterpenoid	Seeds	CH_2_Cl_2_/*n*-Hexane	484	C_26_H_28_O_9_	Bogor, Indonesia	[72]
		Seeds	MeOH/EtOAc	-	-	Pontianak, West Kalimantan, Indonesia	[71]
*Seco*-Dukunolide F (**46**)	Tetranortriterpenoid	Seeds	CH_2_Cl_2_/*n*-Hexane	500	C_27_H_32_O_9_	Nakhon Si Thammarat, Thailand	[73]
		Seeds	MeOH/EtOAc	-	-	Pontianak, West Kalimantan, Indonesia	[71]
Kokosanolide A (**47**)	Tetranortriterpenoid	Seeds	CH_2_Cl_2_/*n*-Hexane	500	C_27_H_32_O_9_	Malaysia	[74]
		Seeds	MeOH/*n*-Hexane	-	-	Cililin, Bandung, Indonesia	[75]
Kokosanolide B (**48**)	Tetranortriterpenoid	Bark	MeOH/*n*-Hexane	456	C_30_H_48_O_3_	Cililin, Bandung, Indonesia	[76]
		Bark	MeOH/EtOAc	-	-	Cililin, Bandung, Indonesia	[75]
		Bark	MeOH/EtOAc	-	-	Cililin, Bandung, Indonesia	[77]
Kokosanolide C (**49**)	Tetranortriterpenoid	Seeds	MeOH/*n*-Hexane	486	C_27_H_34_O_8_	Cililin, Bandung, Indonesia	[75]
Kokosanolide D (**50**)	Tetranortriterpenoid	Fruit peels	MeOH/*n*-BuOH (Kokossan)	516	C_27_H_32_O_10_	Cililin, Bandung, Indonesia	[78]
8,14-Secogammacera-7,14-diene-3,21-dione (**51**)	Tetranortriterpenoid	Bark	MeOH/*n*-Hexane	438	C_30_H_46_O_2_	Cililin, Bandung, Indonesia	[79]
		Bark	MeOH/EtOAc	-	-	Cililin, Bandung, Indonesia	[75]
		Bark	MeOH/EtOAc	-	-	Cililin, Bandung, Indonesia	[77]
		Leaves	MeOH/EtOAc	-	-	Ba’kelalan, Sarawak, Malaysia	[62]
*α*,*γ*-Onoceradienedione = 8,14-Secogammacera-7,14(27)-diene-3,21-dione (**52**)	Onoceranoid triterpenoid	Fruit peels	CH_2_Cl_2_/EtOAc	438	C_30_H_46_O_2_	Laguna, Philippine	[58]
		Bark	EtOH/EtOAc	-	-	Apo Kayan Indonesia	[34,68]
		Bark	MeOH/*n*-Hexane	438	C_30_H_46_O_2_	Cililin, Bandung, Indonesia	[79]
		Fruit peels	MeOH/*n*-Hexane	-	-	Nganjuk, East Java, Indonesia	[80]
24(*E*)-Cyclolanost-24-en-3-one, 21,23-epoxy-21,22-dihydroxy (21*R*,22*S*,23*S*) (**53**)	Cycloartane triterpenoid	Leaves	MeOH/EtOAc (kokossan)	470	C_30_H_46_O_4_	Cililin, Bandung, Indonesia	[81]
		Bark	MeOH/EtOAc	-	-	Cililin, Bandung, Indonesia	[75]
		Leaves	MeOH/EtOAc	-	-	Cililin, Bandung, Indonesia	[77]
3-Oxo-α-bourbonene (**54**)	Sesquiterpene	Fruit peels	-	218	C_15_H_22_O	-	[82]
Stigmasterol (**55**)	Sterol	Peel	Hexane/CH_2_Cl_2_	412	C_29_H_48_O	-	[83]
β-Sitosterol (**56**)	Sterol	Peel	Hexane/CH_2_Cl_2_	414	C_29_H_50_O	-	[83]
4-Hydroxy-N-methylproline (**57**)	Nitrogenous compound	Fruit peels	MeOH	145	C_6_H_11_NO_3_	Bogor, Indonesis	[84]
Domesticulide A (**58**)	Tetranortriterpenoid	Seeds	CH_2_Cl_2_/EtOAc	486	C_27_H_34_O_8_	Thumbon Nopitum, Nakhon Si Thammarat, Thailand	[65]
Domesticulide B (**59**)	Tetranortriterpenoid	Seeds	CH_2_Cl_2_/EtOAc	528	C_29_H_36_O_9_	Thumbon Nopitum, Nakhon Si Thammarat, Thailand	[65]
Domesticulide C (**60**)	Tetranortriterpenoid	Seeds	CH_2_Cl_2_/EtOAc	560	C_29_H_36_O_11_	Thumbon Nopitum, Nakhon Si Thammasat, Thailand	[65]
Domesticulide D (**61**)	Tetranortriterpenoid	Seeds	CH_2_Cl_2_/EtOAc	560	C_29_H_36_O_11_	Thumbon Nopitum, Nakhon Si Thammarat, Thailand	[65]
Domesticulide E (**62**)	Tetranortriterpenoid	Seeds	CH_2_Cl_2_/EtOAc	516	C_27_H_32_O_10_	Thumbon Nopitum, Nakhon Si Thammarat, Thailand	[65]
6-Hydroxymexicanolide (**63**)	Swietenine triterpenoid	Seeds	CH_2_Cl_2_/*n*-Hexane	484	C_27_H_32_O_8_	Thailand	[85]
		Seeds	CH_2_Cl_2_/EtOAc	-	-	Thumbon Nopitum, Nakhon Si Thammarat, Thailand	[65]
6-Acetoxymexicanolide = Ekeberin C_3_ (**64**)	Tetranortriterpenoid	Seeds	CH_2_Cl_2_/EtOAc	526	C_29_H_34_O_9_	Thumbon Nopitum, Nakhon Si Thammarat, Thailand	[65]
		Leaves	EtOH/EtOAc			Menglun town of Yunnan, China	[86]
Methyl angolensate (**65**)	Tetranortriterpenoid	Seeds	CH_2_Cl_2_/EtOAc	470	C_27_H_34_O_7_	Thumbon Nopitum, Nakhon Si Thammarat, Thailand	[65]
Methyl 6-hydroxyangolensate (**66**)	Tetranortriterpenoid	Seeds	CH_2_Cl_2_/EtOAc	486	C_27_H_34_O_8_	Thumbon Nopitum, Nakhon Si Thammarat, Thailand	[65]
Methyl 6-acetoxyangolensate (**67**)	Tetranortriterpenoid	Seeds	CH_2_Cl_2_/EtOAc	528	C_29_H_36_O_9_	Thumbon Nopitum, Nakhon Si Thammarat, Thailand	[65]
Azadiradione (**68**)	Tetranortriterpenoid	Seeds	CH_2_Cl_2_/EtOAc	450	C_28_H_34_O_5_	Thumbon Nopitum, Nakhon Si Thammarat, Thailand	[65]
Onoceratriene (**69**)	Onoceranoid triterpenoid	Bark	EtOH/EtOAc	408	C_30_H_48_	Apo Kayan Indonesis	[34,68]
Lansionic acid = 3-Ketolansiolic acid (**70**)	Onoceranoid triterpenoid	Fruit peels	MeOH/EtOAc	454	C_30_H_46_O_3_	Khon Kaen, Thailand	[61]
		Fruit peels	CH_2_Cl_2_/EtOAc	-	-	Laguna, Philippine	[58]
		Bark	EtOH/EtOAc	-	-	Apo Kayan Indonesis	[34,68]
		Fruit peels	MeOH/EtOAc	-	-	Ra-ngae, Narathiwat, Thailand	[66]
Lansionic acid A = Lansiolic acid A (**71**)	Onoceranoid triterpenoid	Bark	EtOH/EtOAc	470	C_30_H_46_O_4_	Apo Kayan Indonesis	[34,68]
		Leaves	MeOH/EtOAc	-	-	Ba’kelalan, Sarawak, Malaysia	[62]
21*α*-Hydroxyonocera-8(26),14-dien-3-one = 3-keto-22-hydroxyonoceradiene (**72**)	Onoceranoid triterpenoid	Fruit peels	MeOH/EtOAc	440	C_30_H_48_O_2_	Khon Kaen, Thailand	[61]
		Bark	EtOH/EtOAc	-	-	Apo Kayan Indonesis	[34,68]
Methyl lansionate A = methyl lansiolate A (**73**)	Onoceranoid triterpenoid	Bark	EtOH/EtOAc	484	C_31_H_48_O_4_	Apo Kayan Indonesis	[68]
8,14-Secogammacera-14-hydroxy-7-ene-3,21-dione (**74**)	Tetranortriterpenoid	Bark	MeOH/EtOAc	456	C_30_H_48_O_3_	Cililin, Bandung, Indonesia	[75]
Lansium acid I (**75**)	Onoceranoid triterpenoid	Leaves	MeOH/EtOAc	470	C_30_H_46_O_4_	Ba’kelalan, Sarawak, Malaysia	[62]
Lansium acid II (**76**)	Onoceranoid triterpenoid	Leaves	MeOH/EtOAc	486	C_30_H_46_O_5_	Ba’kelalan, Sarawak, Malaysia	[62]
Lansium acid III (**77**)	Onoceranoid triterpenoid	Leaves	MeOH/EtOAc	468	C_30_H_44_O_4_	Ba’kelalan, Sarawak, Malaysia	[62]
Lansium acid IV (**78**)	Onoceranoid triterpenoid	Leaves	MeOH/EtOAc	470	C_30_H_46_O_4_	Ba’kelalan, Sarawak, Malaysia	[62]
Lansium acid V (**79**)	Onoceranoid triterpenoid	Leaves	MeOH/EtOAc	504	C_30_H_48_O_6_	Ba’kelalan, Sarawak, Malaysia	[62]
Lansium acid VI (**80**)	Onoceranoid triterpenoid	Leaves	MeOH/EtOAc	604	C_35_H_56_O_8_	Ba’kelalan, Sarawak, Malaysia	[62]
Lansium acid VII (**81**)	Onoceranoid triterpenoid	Leaves	MeOH/EtOAc	620	C_35_H_56_O_9_	Ba’kelalan, Sarawak, Malaysia	[62]
Lansium acid VIII (**82**)	Onoceranoid triterpenoid	Leaves	MeOH/EtOAc	691	C_38_H_61_NO_10_	Ba’kelalan, Sarawak, Malaysia	[62]
Lansium acid IX (**83**)	Onoceranoid triterpenoid	Leaves	MeOH/EtOAc	620	C_35_H_56_O_9_	Ba’kelalan, Sarawak, Malaysia	[62]
Ethyl lansiolate (**84**)	Onoceranoid triterpenoid	Leaves	MeOH/EtOAc	484	C_32_H_52_O_3_	Ba’kelalan, Sarawak, Malaysia	[62]
Lamesticumin A (**85**)	Onoceranoid triterpenoid	Twigs	EtOH/EtOAc	502	C_31_H_50_O_5_	Xishuangbanna, Mengla, Yunnan, China	[87]
		Leaves	MeOH/EtOAc	-	-	Ba’kelalan, Sarawak, Malaysia	[62]
		Fruit peels	EtOAc/*n*-Hexane	-	-	Bantul, Yogyakarta, Indonesia	[35]
Lansium acid X (**86**)	Onoceranoid triterpenoid	Leaves	MeOH/EtOAc	470	C_30_H_46_O_4_	Ba’kelalan, Sarawak, Malaysia	[88]
Lansium acid XI (**87**)	Onoceranoid triterpenoid	Leaves	MeOH/EtOAc	673	C_38_H_59_NO_9_	Ba’kelalan, Sarawak, Malaysia	[88]
Lansium acid XII (**88**)	Onoceranoid triterpenoid	Leaves	MeOH/EtOAc	604	C_35_H_56_O_8_	Ba’kelalan, Sarawak, Malaysia	[88]
Lansium acid XIII (**89**)	Cycloartane triterpenoid	Leaves	MeOH/EtOAc	470	C_30_H_46_O_44_	Ba’kelalan, Sarawak, Malaysia	[88]
3*β*-Hydroxyonocera-8(26),14-dien-21-one (**90**)	Onoceranoid triterpenoid	Fruit peels	MeOH/EtOAc	440	C_30_H_48_O_2_	Khon Kaen, Thailand	[61]
		Fruit peels	CH_2_Cl_2_/EtOAc	-	-	Laguna, Philippine	[58]
		Fruit peels	MeOH/EtOAc	-	-	Ra-ngae, Narathiwat, Thailand	[66]
3-Hydroxy-8,14-secogammacera-7,14-dien-21-one (**91**)	Onoceranoid triterpenoid	Fruit peels	*n*-Hexane/EtOAc	440	C_30_H_48_O_2_	Cililin, West Java, Indonesia	[89]
3-Oxo-24-cycloarten-21-oic acid (**92**)	Cycloartane triterpenoid	Leaves	MeOH/EtOAc	454	C_30_H_46_O_3_	Indonesia	[67]
Obebioside A (**93**)	Cardenolide	Leaves	MeOH/EtOAc	696	C_36_H_56_O_13_	Thailand	[90]
Obebioside B (**94**)	Cardenolide	Leaves	MeOH/EtOAc	754	C_38_H_58_O_15_	Thailand	[90]
Honghelin (**95**)	Cardenolide	Leaves	MeOH/EtOAc	534	C_30_H_46_O_8_	Thailand	[90]
Obeside B (**96**)	Cardenolide	Leaves	MeOH/EtOAc	592	C_32_H_48_O_10_	Thailand	[90]
Obeside C (**97**)	Cardenolide	Leaves	MeOH/EtOAc	550	C_30_H_46_O_9_	Thailand	[90]
Digitoxigenin (**98**)	Cardenolide	Leaves	MeOH/EtOAc	374	C_23_H_34_O_4_	Thailand	[90]
2-Ethyl,l,3-(2‘-menthene) propenal (**99**)	Sesquiterpene	Fruit peels	EtOAc/*n*-Hexane	220	C_15_H_24_O	Purbalingga, Central Java, Indonesia	[91]
Lamesticumin G (**100**)	Onoceranoid triterpenoid	Fruit peels	MeOH/EtOAc	452	C_30_H_44_O_3_	Ra-ngae, Narathiwat, Thailand	[66]
17(20)*E*-Dyscusin B (**101**)	Pregnane	Leaves	EtOH/EtOAc	330	C_21_H_30_O_3_	Menglun town of Yunnan, China	[86]
17(20)*Z*-Dyscusin B (**102**)	Pregnane	Leaves	EtOH/EtOAc	330	C_21_H_30_O_3_	Menglun town of Yunnan, China	[86]
3-Oxoanticopalic acid methyl ester (**103**)	Diterpene	Leaves	EtOH/EtOAc	332	C_21_H_32_O_3_	Menglun town of Yunnan, China	[86]
(23*R*)-3-Oxo-5α-cycloart-24-en-21,23-olide (**104**)	Cycloartane triterpenoid	Leaves	MeOH/EtOAc	452	C_30_H_44_O_3_	Menglun town of Yunnan, China	[86]
Lansioside D (**105**)	Onoceranoid triterpenoid	Fruit peels	EtOH/Acetone	646	C_37_H_58_O_9_	Laguna, Philippines	[92]
Lamesticumin B (**106**)	Onoceranoid triterpenoid	Twigs	EtOH/EtOAc	488	C_31_H_52_O_4_	Xishuangbanna, Mengla, Yunnan, China	[87]
Lamesticumin C (**107**)	Onoceranoid triterpenoid	Twigs	EtOH/EtOAc	454	C_30_H_46_O_3_	Xishuangbanna, Mengla, Yunnan, China	[87]
Lamesticumin D (**108**)	Onoceranoid triterpenoid	Twigs	EtOH/EtOAc	454	C_30_H_46_O_3_	Xishuangbanna, Mengla, Yunnan, China	[87]
Lamesticumin E (**109**)	Onoceranoid triterpenoid	Twigs	EtOH/EtOAc	484	C_31_H_48_O_4_	Xishuangbanna, Mengla, Yunnan, China	[87]
Lamesticumin F (**110**)	Onoceranoid triterpenoid	Twigs	EtOH/EtOAc	458	C_30_H_50_O_3_	Xishuangbanna, Mengla, Yunnan, China	[87]
Langsatide A (**111**)	Tetranortriterpenoid	Seeds	MeOH/EtOAc	526	C_29_H_34_O_9_	Pontianak, West Kalimantan, Indonesia	[71]
Langsatide B (**112**)	Tetranortriterpenoid	Seeds	MeOH/EtOAc	438	C_26_H_30_O_6_	Pontianak, West Kalimantan, Indonesia	[71]

**Figure 1:**
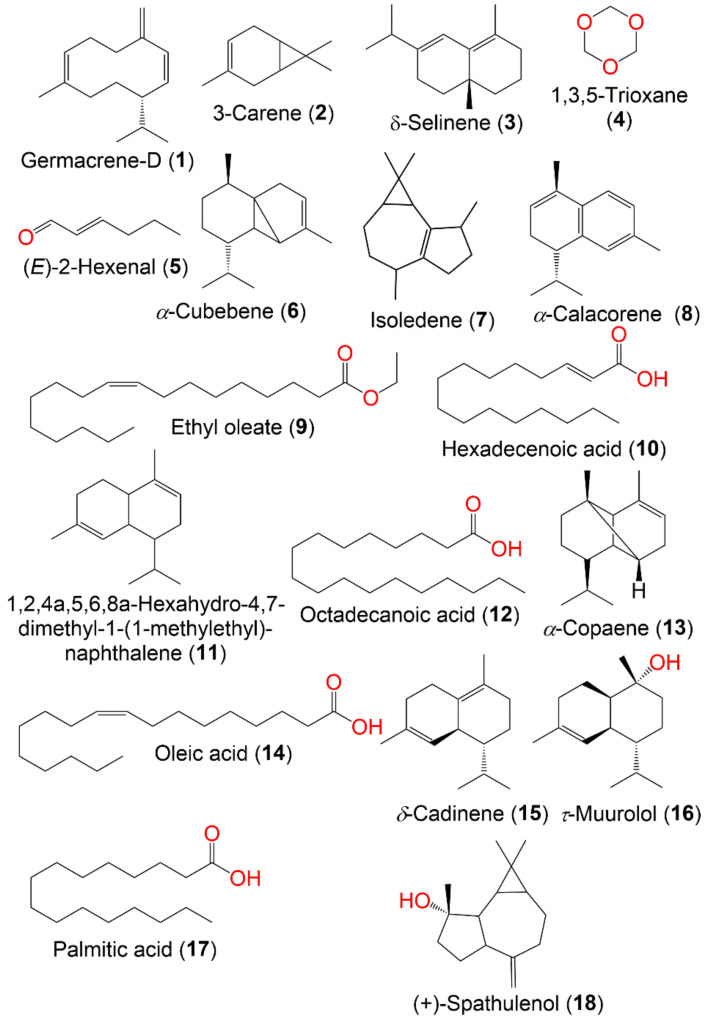
Structures of compounds **1**–**18** from *Lansium domesticum*.

Furthermore, it was reported that the GCMS analysis of the longkong young fruit hot chloroform extract indicated the existence of 10.58% ethyl oleate (**9**), 11.53% hexadecanoic acid (**10**), 6.86% 1,2,4a,5,6,8a-hexahydro-4,7-dimethyl-1-(1-methylethyl)-naphthalene (**11**), 6.05% octadecanoic acid (**12**), and 5.97% α-cubebene (**6**) as dominant constituents [43]. Nevertheless, the GCMS analysis of the essential oil of longkong fruits collected from Narathiwat province showed the presence of α-copaene (**13**) (11.15%), oleic acid (**14**) (14.80%), δ-cadinene (**15**) (6.74%), germacrene-D (**1**) (9.16%), τ-muurolol (**16**) (6.34%), palmitic acid (**17**) (5.49%), and (+) spathulenol (**18**) (5.72%) [57] (Figure 1).

**Figure 2:**
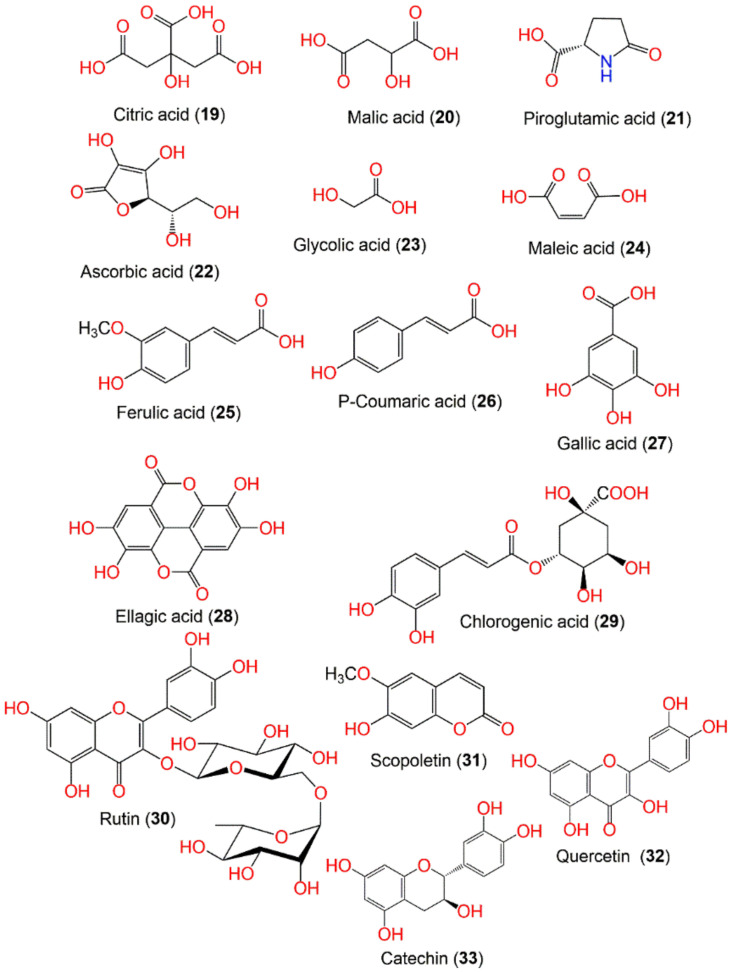
Structures of compounds **19**–**33** from *Lansium domesticum*.

The organic acids and their concentration in langsat and duku fruits were assessed using reversed-phase HPLC technique. The results revealed that the total organic acids in duku and langsat fruits were 0.604 and 1.04%, respectively, where citric (**19**) and malic acids (**20**) represented the major acids found in both fruits. Whilst piroglutamic (**21**) and ascorbic (**22**) acids existed in low concentrations [36]. Moreover, citric acid (**19**), glycolic acid (**23**), maleic acid (**24**), and malic acid (**20**) are the predominant acids found in the fruit (Figure 2) [57].

### 7.2. Phenolics

It was stated that the longkong peel and flesh had a high phenolic content that is affected by the initiation of the phenylalanine ammonia-lyase activity upon external stimuli leading to abundant phenolics production [12]. Ferulic (**25**), p-coumaric (**26**), and gallic (**27**) acids, ellagic acid (**28**), and a high level of tannins were reported in longkong fruit [20,59,93]. Further, the phytochemical analysis of the ethyl acetate (LDSK50-EA) and aqueous (LDSK50-H_2_O) fractions of longkong peels illustrated the presence of phenolics, mainly chlorogenic acid (**29**), rutin (**30**), and scopoletin (**31**) [51]. It is noteworthy to state that the pericarp possessed a higher flavonoid content than flesh, while the seeds have no flavonoids [53]. A high flavonoids yield was observed in the fruit extracted with hot H_2_O in comparison to other kinds of solvents [43]. Alimon et al., reported the presence of flavonoids in langsat, duku, and longkong [93]. It is noteworthy that many flavonoids are found in the fruit, however, only rutin (**30**), quercetin (**32**), and catechin (**33**) have been detected (Figure 2) [43,51].

### 7.3. Terpenoids

**Figure 3:**
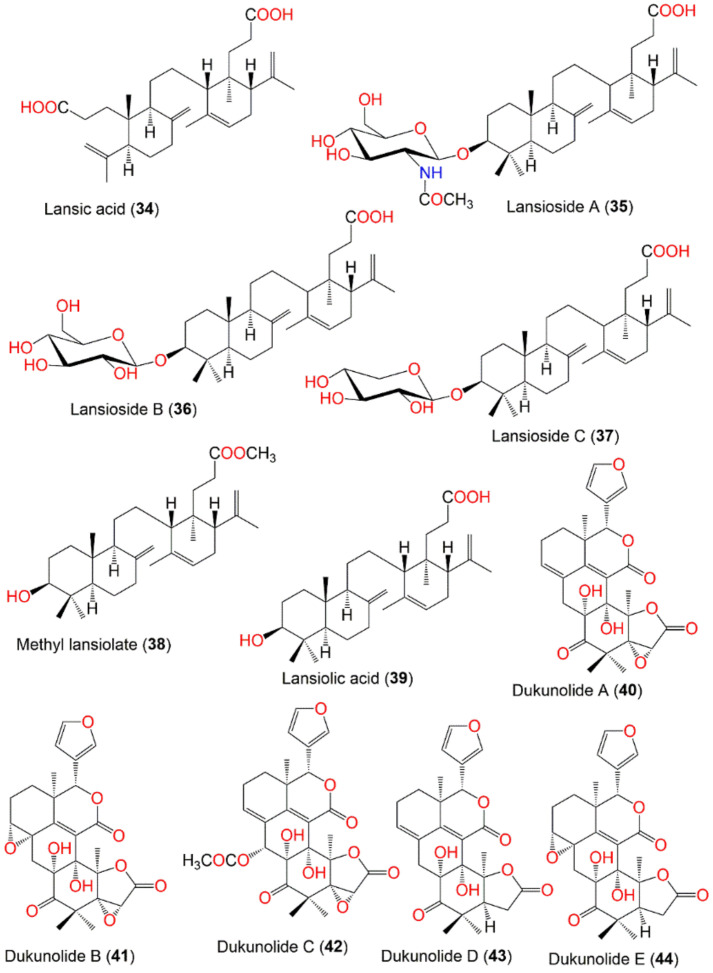
Structures of compounds **34**–**44** from *Lansium domesticum*.

The peel was reported to contain a large quantity of latex that had lansic acid (**34**) as a major component of the latex that was isolated firstly in 1967 by Kiang et al., from the light petroleum peel extract [94]. Lansioside A (**35**), a novel seco-onoceran aminoglucoside triterpenoid was isolated from the EtOH extract of *L. domesticum* peel by SiO_2_ CC. It had an acetyl group linked to the nitrogen atom, characterizing the existence of *N*-acetyl-D-glucosamine. Its configuration was established by NMR and chemical derivation, as well as optical rotation [60,63]. In another study, Nishizawa et al., obtained seco-onoceran triterpenoids; lansic acid (**34**) and lansiosides B and C (**36** and **37**) from the peel CH_2_Cl_2_ fraction by SiO_2_ CC. They gave the same aglycone methyl ester, methyl lansiolate (**38**) on methanolysis (Figure 3) [63]. Lansiosides B and C (**36** and **37**) are β-D-glucopyranoside and β-D-xyloside, respectively [19]. Compound **35** was found to inhibit leukotriene D_4_-induced contraction of guinea pig ileum in vitro in a dose-dependent way (IC_50_ 2.4 × l0^6^ g/mL, 2.4 ppm), while **36** and **37** were 10-fold less potent and **39** was inactive [19].

Dukunolide A (**40**), a tetranortriterpenoid with a novel 26-carbon skeleton was purified from n-hexane extract of duku seeds by SiO_2_ CC and recrystallization. Its structure was established by NMR spectroscopic data and single-crystal X-ray diffraction [69]. Nishizawa et al., isolated and characterized dukunolides A (**40**), B (**41**), and C (**42**), as well as revising the structure of **40** using NMR; the absolute configuration was deduced by chemical method and X-ray analysis. Compound **40** possessed a UV bathochromic shift due to the α,β,γ,δ-dienolide system and *cis*- ring junctions at C-l/C-2 and C-5/C-10, whereas **41** had a C-8/C-9 epoxide and saturated doubly conjugated δ-lactone moieties at the γ,δ-positions. Compound **42** was similar to **40**, with an additional secondary acetoxyl group at C-22 [70]. Further, the same authors in 1988 isolated dukunolides D-F (**43**–**45**) from the CH_2_Cl_2_ extract of by SiO_2_ CC using CH_2_Cl_2_/n-hexane or CH_2_Cl_2_/EtOAc as solvent system. Their structures were elucidated by NMR and the absolute configuration was deduced by X-ray analysis [72]. Dukunolides D (**43**) and E (**44**) were structurally similar to **40** and **41**, respectively, with the absence of the 5,6-oxirane ring. Whilst dukunolide F (**45**) was assigned as stereoisomer of **44** [72] (Figure 3).

**Figure 4:**
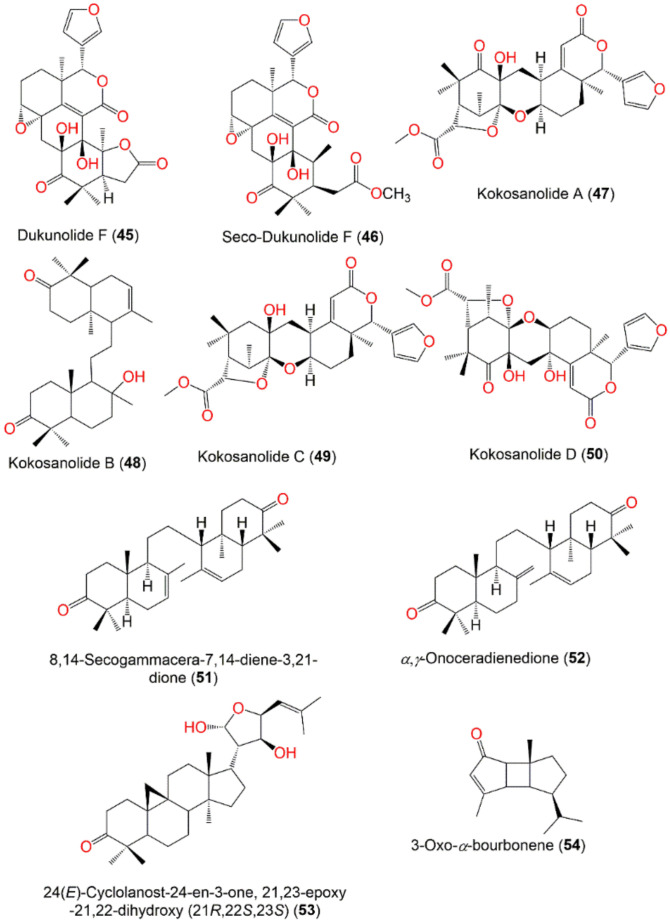
Structures of compounds **45**–**54** from *Lansium domesticum*.

*Seco*-Dukunolide F (**46**), a 4-ring A/B/C/D fused tetranortriterpenoid was obtained from the seeds CH_2_Cl_2_ extract of Thai by SiO_2_ CC using CH_2_Cl_2_/n-hexane or CH_2_Cl_2_/EtOAc as an eluting system. This compound possessed no antimalarial, antitubercular or anti-tumor activities [73]. Kokosanolide A (**47**), a tetranortriterpenoid was isolated from the *n*-hexane fraction of the Malaysian *L. domestlcum* seeds by SiO_2_ CC and characterized by NMR and crystallographic analyses [74] (Figure 4). Supratman et al., obtained kokosanolide B (**48**) as crystals by SiO_2_ CC from the bark EtOAc fraction. This compound structurally resembled **47**, however, an H_2_O was added to the endocyclic double bond to provide the corresponding alcohol [76]. Whilst kokosanolide D (**50**) was obtained from the peels n-BuOH fraction elucidated using IR, NMR, and HRMS [78].

Tjokronegero et al., isolated 2 new tetranortriterpenoids; 8,14-secogammacera-7,14-diene-3,21-dione (**51**) and α,γ-onoceradienedione (**52**) from the *n*-hexane fraction of the bark using SiO_2_ CC and PTLC (preparative thin-layer chromatography). These metabolites possessed two fused tetrahydrodecalin-type rings linked through an ethylene group. Their structure was assigned based on NMR and crystallographic techniques [79].

The EtOAc extract of *L. domesticum* leaves afforded a new cycloartan-type triterpenoid, 24(*E*)-cyclolanost-24-en-3-one, 21,23-epoxy-21,22-dihydroxy (21*R*, 22*S*, 23*S*) (**53**) that was purified by repeated SiO_2_ CC and recrystallization in acetone. Its structure was elucidated by NMR and X-ray diffraction. It was characterized by a furan ring at C-20, C-21, C-22, and C-23, respectively [81].

Uyehara et al., established that cis-cisoid-cis isomer of 3-oxo-α-bourbonene (**54**) that was reported as toxic fish poison from *L. domesticum* had a unique cisoid-(5–4–5) fused ring skeleton and it was not identical to the toxic components of *L. domesticum* [82] (Figure 4).

### 7.4. Sterols

**Figure 5:**
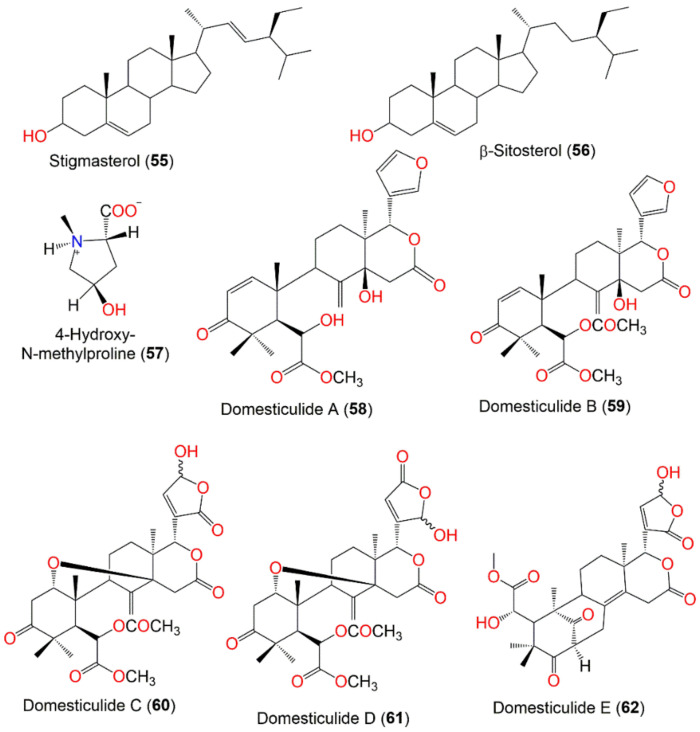
Structures of compounds **55**–**62** from *Lansium domesticum*.

The fruit and its parts featured very few phytosterols. Pooasa isolated stigmasterol (**55**) and β-sitosterol (**56**) from the peel hexane and CH_2_Cl_2_ extracts. Moreover, the existence of triterpenes and unsaturated sterols was also reported [24,83] (Figure 5).

## 8. Biological Activities of *L. domesticum* Extracts and Isolated Compounds

### 8.1. Anti-Malarial Activity

Malaria is a serious parasitic disease in tropical and subtropical regions all over the world, with 435,000 deaths and 219 million infections cited in 2017 [95]. The incidence of malaria has re-emerged in part due to several strains of *P. falciparum* becoming resistant to the available antimalarial agents. Thence, there is a crucial need for discovering new anti-malarial agents and for verifying the safety and efficiency of traditional medicinal plants that are utilized to fight this disease [96].

*L. domesticum* seeds and bark are traditionally known to be effective towards malaria parasite [30]. *L. domesticum* bark extracts were assessed for in vitro anti-plasmodial potential against chloroquine-resistant clone (W2) and -sensitive *P*. *falciparum* clone (D6). The bark extracts (Conc. 20 µg/mL) were notably active towards chloroquine-resistant clone W2 and exhibited selective potential towards chloroquine-sensitive *P*. *falciparum* clone D6 in the Kenyah malaria [30]. Further, the bark EtOAc fraction had a promising activity towards D6 and W2 *P. falciparum* clones (IC_50_ 3.45 and 5.61 µg/mL, respectively). On the other hand, it had no significant effect on parasite clearance on the *P. bergheii*-infected mice [68]. On the other hand, the skin and leaf aqueous extracts equally reduced parasite number of both drug-sensitive (3D7) and chloroquine-resistant (T9) *P. falciparum*. The skin extracts interrupted the parasite lifecycle, which proved the effectiveness of *L. domesticum* as a source of antimalarial agents towards *P. falciparum* chloroquine-resistant strains [17]. Additionally, the seeds CH_2_Cl_2_ extract was found to significantly prohibit *P. falciparum* (IC_50_ 9.9 µg/mL) [65,85]. It was stated that lansiolic acid (**39**) had antimalarial potential [97]. Yapp et al., obtained 4-hydroxy-N-methylproline (**57**), a cyclic hydroxy-amino acid with *trans* carboxyl and hydroxyl groups as a crystal from the peel MeOH extract. This compound exhibited antimalarial potential towards chloroquine-resistant *P. falciparum* (T9) strain only at concentration >1.0 mg/mL [84].

**Figure 6:**
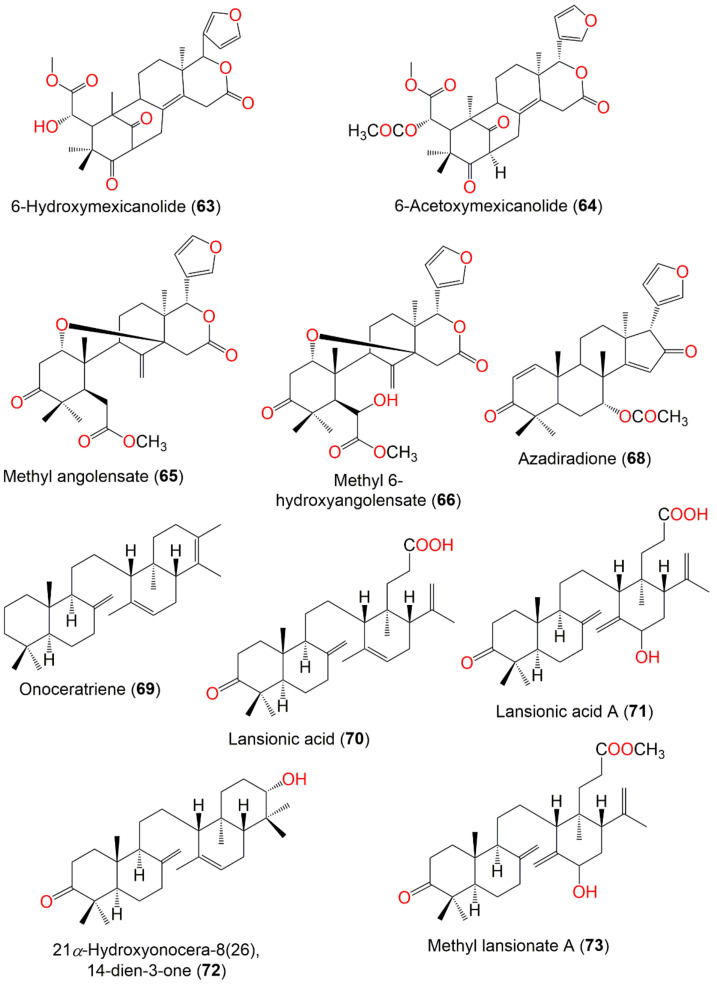
Structures of compounds **63**–**73** from *Lansium domesticum*.

Saewan et al., reported the separation of new tetranortriterpenoids; domesticulides A–E (**58**–**62**), along with 11 known analogs; lansioside B (**36**), lansiolic acid (**39**), and dukunolide C (**42**), 6-hydroxymexicanolide (**63**), 6-acetoxymexicanolide (**64**), methyl angolensate (**65**), methyl 6-hydroxyangolensate (**66**), methyl 6-acetoxyangolensate (**67**), and azadiradione (**68**) from the seeds CH_2_Cl_2_ extract using SiO_2_ CC and preparative TLC [65] (Figure 5 and Figure 6). Compounds **42**, **59–61**, **64**, **65**, **67**, and **68** were moderately active (IC_50_ 2.4–9.7 µg/mL) against *P*. *falciparum* (K1, multidrug-resistant strain), compared to artemisinin (IC_50_ 0.001–0.003 µg/mL) in the microculture radioisotope assay. The results revealed that the C6-hydroxyl group lessened the activity as in **66** (IC_50_ > 20.0 µg/mL), however, the substitution of C6-hydroxyl group with an acetoxy group increased the activity as in **59** (IC_50_ 3.2 µg/mL), **63** (IC_50_ 9.7 µg/mL), and **67** (IC_50_ 3.8 µg/mL). The most active compounds were **59**, **60**, **67**, and **68** (IC_50_ values of 3.2, 2.4, 3.8, and 2.9 µg/mL, respectively) [65].

Moreover, 6-Hydroxymexicanolide (**63**) had a swietenine skeleton (A/B/C/D 4-fused ring triterpenoid system) was purified from the seeds CH_2_Cl_2_ extract by SiO_2_ CC using CH_2_Cl_2_/n-hexane or CH_2_Cl_2_/EtOAc and assigned based on NMR and X-ray techniques. It showed no noticeable effect (IC_50_ >20 µg/mL) towards *P. falciparum* [85].

Omar obtained methyl lansiolate (**38**), lansiolic acid (**39**), *α*,*γ*-onoceradienedione (**52**), onoceratriene (**69**), lansionic acid (**70**), lansionic acid A (**71**), 21*α*-hydroxyonocera-8(26),14-dien-3-one (**72**), and methyl lansionate A (**73**) from the bark and assessed for their in vitro antimalarial potential towards the chloroquine-sensitive (D6) and chloroquine-resistant (W2) *P. falciparum*. Compounds **38**, **52**, **72**, and **73** were the most potent compounds towards D6 *P. falciparum* (IC_50_ ranging from 0.65–2.41µg/mL) in comparison to artemisinin (IC_50_ 0.0015 µg/mL) and chloroquine (IC_50_ 0.0045 µg/mL), while, only **38**, **52**, and **73** exhibited activity towards W2 *P. falciparum* (IC_50_ values of 0.76, 1.83, and 1.02 µg/mL, respectively), compared to artemisinin (IC_50_ 0.0035 µg/mL) and chloroquine (IC_50_ 0.0065 µg/mL) [68] (Figure 6). Besides, **38** and **52** (Conc. 50 mg/kg/day) suppressed parasitemia levels by 44 and 20%, respectively, in the *P*. *bergheii*-infected mice, compared to quinine (dose of 10 mg/kg, 60%). Whilst **72** and **73** had no significant effect of parasite clearance on the infected mice [68] (Table 5).

### 8.2. Antifeedant, Insecticidal, and Larvicidal Activities

Natural pest controlling agents have been publicized as substitutes to synthetic chemicals for integrated pest management. These phytochemicals are known to pose little threat to human health or to the environment [99]. Recently, there has been a fast-growing interest in the use of more ecologically acceptable methods to protect the food supply from predatory insect attacks [100]. Antifeedants are compounds that either prevent insect feeding (feeding deterrent effect) or cause slowing or cessation of further feeding (feeding suppressant effect) [99,101]. They attract special attention owing to their potential utilization in integrated pest control systems [102].

**Figure 7:**
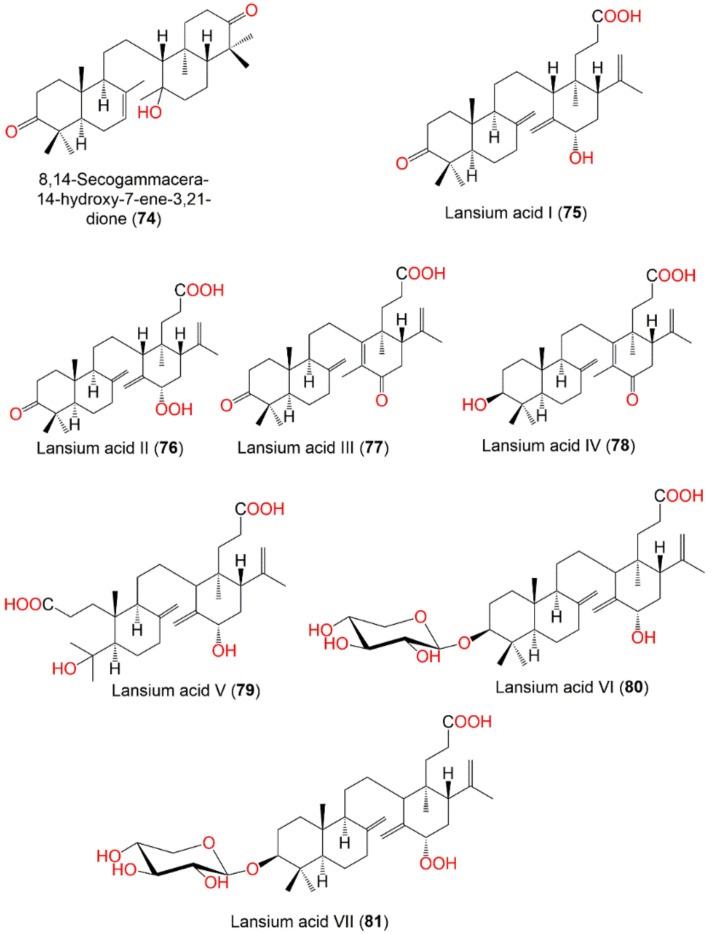
Structures of compounds **74**–**81** from *Lansium domesticum*.

The feeding deterrence study towards *Sitophilus oryzae* revealed that flour disks prepared using the barks ethyl acetate and n-hexane fractions totally inhibited the diet consumption at 0.50% (*w*/*w*), but the H_2_O extract was phagostimulatory [34]. Mayanti et al., isolated two tetranortriterpenoids; kokosanolide A (**47**) and C (**49**) and 3 onoceranoid-triterpenoids; kokosanolide B (**48**), 8,14-secogammacera-7,14-diene-3,21-dione (**51**), and 8,14-secogammacera-7,14(27)-diene-3,21-dione (**52)** from the seeds n-hexane fraction and barks EtOAc fraction, respectively [75]. They possessed moderate to potent antifeedant activity with 78, 0, 99, 85, and 56%, respectively, towards the fourth instar larvae of *Epilachna vigintioctopunctata* (Conc. 1%) [75] (Figure 7). Compounds **39**, **52**, **69**, **71**, and **72** isolated from the bark EtOAc fraction exhibited significant insect antifeedant potential towards *Sitophilus oryzae* (rice weevil) using a flour disk bioassay (Conc. 0.5% *w*/*w*), however, **70** was inactive at this concentration. Compounds **52**, **71**, and **72** had the highest activity with % consumption of diet 40.1, 56.1, and 53.8%, respectively [34,68]. Arnason et al., also reported the insect feeding deterrent potential of **39** [97].

Leatemia et al., stated that the EtOH seed extracts of *L. domesticum* obtained from different locations and years in Maluku, Indonesia had an insecticidal potential towards *Spodoptera litura*, with a % growth inhibition of 78–118% with no significant effect of collection locations on the activity [103]. Additionally, the aqueous extract of fresh leaves was evaluated for larvicidal potential towards *Aedes aegvpti* and *Culex quinquefasciatus* by exposing 3rd–4th instar larvae to different concentrations of the extract. The extract was highly effective towards larvae of *Ae. aegvpti* (LD_50_ and LD_90_ 4.0847 and 37.7165 g%) and *Cx. quinquejbsciatus* (LD_50_ and LD_90_ 4.0289 and 16.3316 g%) [32].

### 8.3. Anti-Fertility Activity

The increase in the human population is one of the most critical problems throughout the world, especially in underdeveloped and developing countries [104]. The evaluation of the antifertility potential of the medicinal plant has been growing worldwide as a means of identifying safe and effective agents for controlling the population explosion [105]. *L. domesticum* bark water decoction was used by rural communities in East Kalimantan as an anti-fertility agent. The potential of water decoction of bark stew on uterus weight and estrous cycle in mice had been assessed [31]. The estrous cycle is the reproductive cycle in female mice that ranges from 4–5 days. The results revealed that H_2_O decoction of the bark had no remarkable effect on the uterine weight and estrous cycle in female mice. Therefore, the anti-fertility potential of the bark H_2_O decoction was not proven [31].

### 8.4. Antimutagenic Activity

Mutagens are agents that can invoke mutations [106]. They are not only included in carcinogenesis and genotoxicity but also the pathogenesis and inception of many chronic diseases, including neurodegenerative, cardiovascular, and hepatic disorders, chronic inflammation, arthritis, diabetes, and aging [107,108]. Natural antimutagenics are known to protect against the detrimental effects of mutagens. They include various plants and their active metabolites such as flavonoids, phenolics, coumarins, carotenoids, tannins, anthraquinones, saponins, and terpenoids [107].

The MeOH extract of leaves exhibited antimutagenic potential towards 2-amino-1-methyl-6-phenylimidazo (4,5-b)pyridine (PhIP) and 3-amino-1,4-dimethyl-5H-pyrido [4,3-b]indole (Trp-P-1)-produced mutagenicity with inhibition 80.8 and 75.7%, respectively, at 125 μg/plate in the Ames test [62]. Additionally, the peel 50% EtOAc fraction showed significant anti-mutagenic potential towards mitomycin C-induced mutagenicity in TK6 human lymphoblasts in the cytokinesis-blocked micronucleus assay [109].

**Figure 8:**
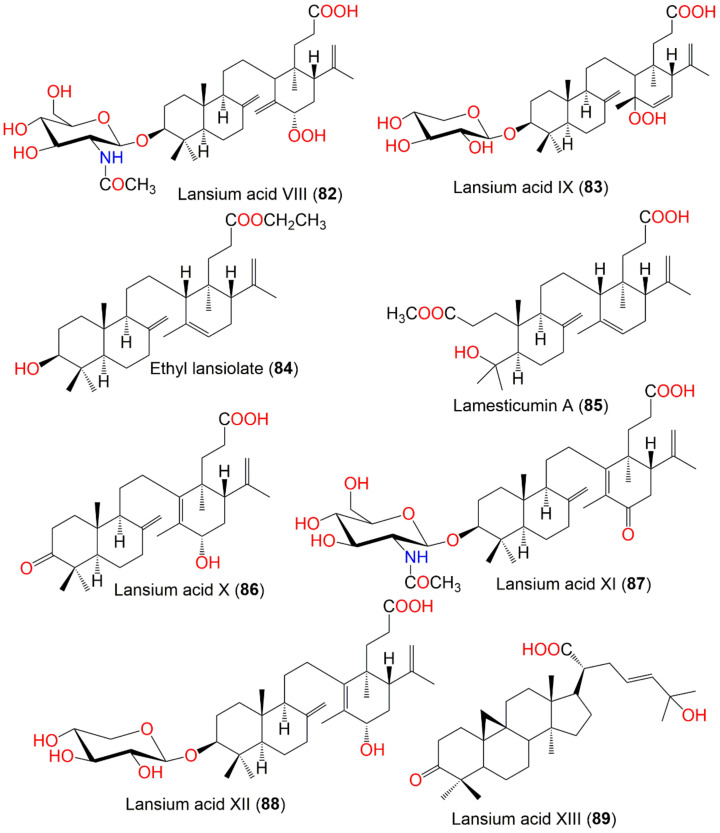
Structures of compounds **82**–**89** from *Lansium domesticum*.

The new onoceranoid triterpenoids, lansium acids I–IX (**75**–**83**), along with **34**, **36**–**39**, **51**, **70**, **84**, and **85**, were purified from the leaves EtOAc fraction using normal- and RP_18_ SiO_2_ CC and repeated HPLC (Figure 8). They were characterized by chemical derivatization and spectroscopic analyses and absolute stereo-structures were assigned via X-ray diffraction and electronic circular dichroism spectra. Compounds **34**, **38**, **39**, and **84** displayed antimutagenic potential towards Trp-P-1 and PhIP in the Ames assay. Further, the oral intake of **70** (Conc. 0.03% or 0.06%, *w*/*w*) exhibited in vivo antimutagenic potential towards PhIP in the micronucleus test. This was evident by the presence of fewer MNRETs (micro-nucleated reticulocytes) caused by PhIP [62].

In 2019, Matsumoto et al., isolated 3 new onoceranoid triterpenoids; lansium acids X–XII (**86**–**88**) and a new cycloartanoid; lansium acid XIII (**89**) (Figure 8). Compounds **86**–**88** had antimutagenic effectiveness towards Trp-P-1 without antimicrobial activity using *Salmonella typhimurium* TA98 strain in the Ames assay. Their effects were attributed to the inhibition of CYP1A2 (cytochrome P450 1A2), which bioactivated Trp-P-1 mutagenicity [88].

### 8.5. Cytotoxic Activity

Cancer represents one of the major reasons for death globally [110]. Many of the available chemotherapies possess serious side effects, drug resistance, and none target specificity [111]. Thus, there is an emerging search to develop drugs from natural sources in order to overcome these drawbacks. Natural metabolites from diverse sources, including microorganisms, plants, and animals, present a great pool for the discovery of novel therapeutic candidates for treating this disease [112].

Recently, it was reported that the n-hexane fraction of the fruit peels demonstrated noticeable activity towards T47D cell lines (IC_50_ 0.1 µg/mL) compared to doxorubicin (IC_50_ 0.04 µg/mL), as well as weak cytotoxic potential towards HeLa and A549 (IC_50_ 59.55 and 18.83 µg/mL, respectively) in the MTT assay [80]. Besides, the peels total EtOAc extract, *n*-hexane soluble fraction, and *n*-hexane insoluble fraction had cytotoxic activity in the MTT assay towards T-47D cancer cell line (IC_50_ 29.41, 43.51, and 25.57 μg/mL, respectively) compared to doxorubicin (IC_50_ 0.18 μg/mL) [91]. The 24, 48, and 72 h-treatment of HT-29 cell with peels MeOH, EtOH, and EtOAc extracts (Conc. 0–100 µg/mL) in the MTT assay revealed that MeOH extract exhibited cytotoxic potential (IC_50_ 6.79 µg/mL) and induced morphological changes towards HT-29 cells line after 27 h, while EtOAc (IC_50_ 86.00 µg/mL) and EtOH extracts displayed a weak or no activity [113].

Moreover, the peel MeOH extract had toxicity against *Artemia salina* [61]. Further, the leaves MeOH extract (Conc. 200 μg/mL) exhibited Notch inhibitory potential by reducing luciferase activity to 30% and cell viability to 62% compared to those of the control [90].

Manosroi et al., assessed the cytotoxic capacities of cold and hot H_2_O, cold and hot MeOH, and cold and hot CHCl_3_ extracts of eight *L. domesticum* parts (young fruits (YF), ripe fruits (RF), old leaves (OL), seeds (SE), young leaves (YL), peels (PE), stalk (ST), and branches (BR)] that were collected from three provinces (Satun, Narathiwat, and Yala) in the south of Thailand towards B_16_F_10_, KB, HepG2, and HT-29 using SRB assay. It is noteworthy that ripe fruits cold water extract (RFWC) had the highest percentage yield (59.38%). The hot and cold MeOH extract of stalks (STMH and STMC) showed the highest total flavonoid and phenolic contents. The young fruit cold (YFCC) and hot CHCl_3_ (YFCH) extracts possessed cytotoxic potential (IC_50_ < 1 mg/mL) towards all cancer cells. In apoptotic induction, YFCH displayed the highest apoptotic effectiveness towards KB with 13.84% at 0.5 mg/mL and towards HT-29 with 8.68% at 5 mg/mL. On the other hand, YFCC had the highest apoptotic potential towards KB cells (10.70% at 0.5 mg/mL) [43]. The YFCH exhibited the highest necrotic induction potential towards KB and B_16_F_10_ cell lines (% necrosis 6.19 and 27.58% at 5 mg/mL, respectively) whereas YFCC had the highest potential towards KB, HT-29, HepG2, and B_16_F_10_ cell lines at 5 mg/mL (% necrosis 45.36, 41.13, and 100%, respectively) [43].

Besides, Manosroi et al., stated that the young fruit hot (NYFCH) and cold chloroform (NYFCC) extracts from the Northern region exhibited antiproliferative effect towards KB cells (IC_50_ 603.45 and 765.06 µg/mL, respectively) in the SRB assay, compared to cisplatin (IC_50_ 12.72 µg/mL), fluorouracil (IC_50_ 12.94 µg/mL), doxorubicin (IC_50_ 0.82 µg/mL), and vincristine (IC_50_ 0.03 µg/mL). The triterpenoids in the chloroform extracts may be accountable for this effect [53]. Additionally, they had higher active MMP-2 inhibitory potential (53.03 and 31.30% for NYFCC and 49.40 and 21.72% for NYFCH) than all anticancer agents except cisplatin. The antioxidative triterpenes in hot chloroform extract inhibited matrix metalloproteases (MMPs), which regulate invasion and cellular motility of cancer cells, indicating that the NYFCH could be further developed to an oral anticancer agent [53].

**Figure 9:**
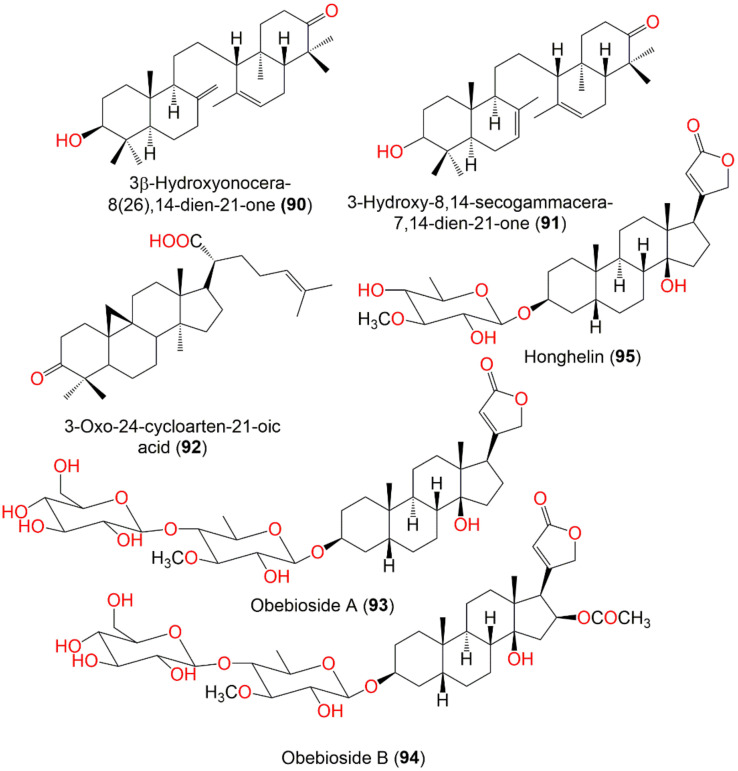
Structures of compounds **90**–**95** from *Lansium domesticum*.

In addition, kokosanolide A (**47**) had potent cytotoxic potential (IC_50_ 8.62 μg/mL) towards MCF-7 cancer cells. Furthermore, the molecular docking study revealed that **47** and **49** showed strong bond-free energy (−8.8 kcal/mol and -8.7 kcal/mol, respectively) to estrogen receptor-α (ERα), therefore they inhibited ERα in breast cancer cells [98]. Tanaka et al., obtained 3 new onoceranoid triterpenes; **70**, **72**, and **90** from the peel EtOAc fraction that exhibited moderate toxicity against *A. salina* (Conc. 100 µg/mL) [61] (Figure 9).

Putri et al., separated 3-hydroxy-8,14-secogammacera-7,14-dien-21-one (**91**), a new onoceranoid triterpenoid from peel n-hexane extract that exhibited weak activity against MCF-7 (IC_50_ 717.5 µM) compared to doxorubicin (IC_50_ 35.7 µM) in the MTT [89].

Additionally, **52** demonstrated weak cytotoxic potential towards A549 (IC_50_ 13.71 μg/mL) and moderate activity towards HeLa and T47D cell lines (IC_50_s 32.32 and 30.69 μg/mL, respectively) compared to doxorubicin (IC_50_ 2.83 and 0.04 μg/mL, respectively) in the MTT assay [80]. Additionally, lamesticumin A (**85**), an onoceranoid-type triterpenoid was isolated from the peels n-hexane fraction that possessed cytotoxic potential towards T-47D (IC_50_ 15.68 µg/mL) compared to doxorubicin (IC_50_ 0.18 µg/mL) in the MTT assay [91].

Nishizawa et al., obtained 3-oxo-24-cycloarten-21-oic acid (**92**), a new cycloartanoid triterpene carboxylic acid, along with **34**, **39**, and **40** from *L. domesticum* leaves. Compound **92** exhibited significant inhibitory activity of skin-tumor promotion at a concentration of 3.2 nM in EBV-EA (Epstein Barr virus activation-early antigen) in Raji cells induced by TPA (12-*O*-tetradecanoyl-phorbol-13-acetate) [67] (Figure 9).

**Figure 10:**
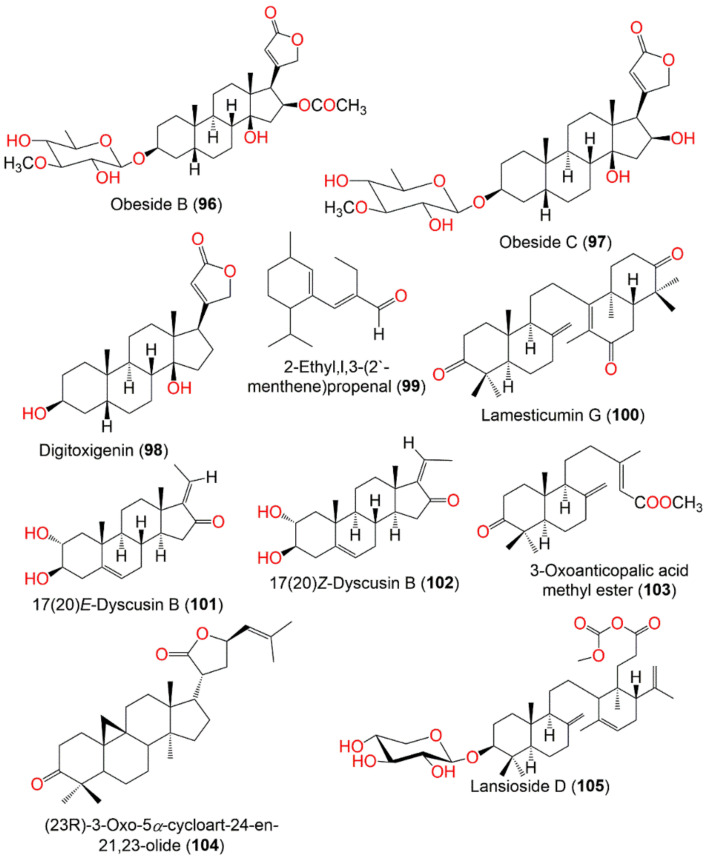
Structures of compounds **96**–**105** from *Lansium domesticum*.

Tsuchiya et al., purified 6 cardenolides, obebioside A (**93**), obebioside B (**94**), honghelin (**95**), obeside B (**96**), obeside C (**97**), and digitoxigenin (**98**) from the EtOAc-soluble fraction of *L. domesticum* leaves by SiO_2_, ODS (octadecyl silica), and ODS-HPLC CC (Figure 10). They were identified by NMR, ESIMS, and optical rotation. These compounds were assessed for their Notch signaling inhibitory potential compared to the DAPT (γ-secretase inhibitor, *N*-(*N*-(3,5-difluorophenacetyl)-l-alanyl)-*S*-phenylglycine *t*-butyl ester). Compounds **93**, **94**, and **95** demonstrated potent Notch inhibition (IC_50_ 1.65, 0.62, and 0.51 μM, respectively), whereas **96**, **97**, and **98** were inactive. Further, **95** was also potent cytotoxic (IC_50_ of 34 nM) towards HPB-ALL in the Alamar Blue assay. It induced the C17.2 neural stem cells differentiation to neurons, resulting in a 65% rise in differentiation. It inhibited Notch signaling through a dual mechanism, including lowering of both MAML (mastermind-like) protein and Notch1 levels [90]. Notch signaling possesses substantial roles in cell differentiation and proliferation, abnormal activation of this signaling promotes cancer progression [114,115]. Therefore, **95** as a Notch signaling inhibitor may be a candidate for an anticancer agent or could have application in neural regenerative medicine [90]. 2-Ethyl,l,3-(2′-menthene)propenal (**99**), an aldehyde sesquiterpene was obtained from the peels EtOAc extract that gave a red-brown color after Ce(SO4)_2_ visualization (Figure 10). It displayed cytotoxic potential towards T-47D (IC_50_ 48.58 µg/mL) and HepG2 (IC_50_ 127.45 µg/mL) compared to doxorubicin (IC_50_ 0.43 and 1.18 µg/mL, respectively) in the MTT assay [35].

### 8.6. Antioxidant Activity

Chronic illnesses such as diabetes, cancer, and cardiovascular and neurodegenerative diseases are featured by an incremented state of oxidative stress that may result from a decline in antioxidant defenses and/or reactive species (ROS) overproduction [116]. Natural compounds are known to have better antioxidant potential than synthetic antioxidants, making them an extremely attractive ingredient for commercial foods [117]. Despite the huge number of natural antioxidative agents, searching for new chemical entities with antioxidant potential remains a growing field.

Klungsupya et al., reported that the peel 50% EtOAc fraction possessed potent antioxidant capacity [109]. Also, the EtOH-EtOAc (50:50%, *v*/*v*) and EtOH:H_2_O (50:50%, *v*/*v*) fractions showed potent O_2_^-*bullet*^ and OH*^bullet^* scavenging activity in the photo-chemiluminescence assay. They had protective potential on H_2_O_2_-induced DNA damage on TK6 human lymphoblast cells (Conc. 25, 50, 100, and 200 μg/mL) in the comet assay [51].

Subandrate et al., reported that the seed extract (dose 100 mg/kg BW) had an antioxidant potential, where it increased GSH (glutathione) and lowered MDA (malondialdehyde) in alcohol-induced rats, therefore it prohibited free radicals and inhibited lipid peroxidation [118]. Moreover, the EtOAc fraction of the seeds exhibited a strong antiradical potential (IC_50_ 8.938 µg/mL) than water fraction, n-hexane fraction, and methanol extract (IC_50_ 13.898, 11.012, and 14.624 µg/mL, respectively) in comparison to vitamin C (IC_50_ 4.721 µg/mL). This effect was referred to its high phenolic and flavonoid contents (58.25 mg GAE/g and 75.123 mg QE/g, respectively) [119].

Manosroi et al., collected various parts of *L. domesticum* from Eastern and Northern Thailand and extracted them by the cold and hot methods using H_2_O, CHCl_3_, and MeOH. The hot seeds H_2_O extract from the Northern region (NSEWH) possessed the highest free radical scavenging (FRS) potential (SC_50_ 0.34 µg/mL) in the DPPH assay, compared to ascorbic acid (SC_50_ 0.08 µg/mL). On the other hand, the hot CHCl_3_ extract of the young leaves from the Eastern region (EYLCH) had the potent lipid peroxidation inhibition (IPC_50_ 0.86 µg/mL), compared to α-tocopherol (IPC_50_ 0.03 µg/mL) in the modified ferric-thiocyanate method. Additionally, the cold-H_2_O extract of the old leaves from the Northern region (NOLWC) exhibited the powerful metal ion chelating potential (MC_50_ 0.47 µg/mL), compared to EDTA (MC_50_ 0.06 µg/mL) in the ferrous ion chelating method. It is noteworthy that the extracts from the Northern region had higher FRS, metal ion chelating, and lipid peroxidation inhibition activity than those from the Eastern region. This might be attributed to the flavonoid and phenolic compounds in the extracts [53].

Apridamayanti et al., reported that the stem bark displayed weak antioxidant potential (IC_50_ 2820 ppm) in the DPPH assay [120].

The 96% EtOH and EtOAc extracts from fruit peels (FP) and flesh (FF) of *L. domesticum* were prepared by maceration with ethyl acetate and 96% ethanol. FP-EtOAc and FF-EtOAc extracts had potent antioxidant potential in the DPPH, BCB (β-carotene bleaching), and FRAP (ferric reducing antioxidant power) assays, respectively [121].

### 8.7. α-Glucosidase Inhibitory Activity

Diabetes continues to be a main health concern worldwide. It is featured by a defect in insulin action and/or secretion associated by hyperglycemia and disruption in lipid, carbohydrate, and protein metabolism [122,123]. The best therapeutic strategy for type-II diabetes is to lower hyperglycemia through retardation of the intake of glucose by repression of α-glucosidases and α-amylases, which are accountable for the di- and oligosaccharides breakdown into glucose [123].

A novel onoceranoid triterpenoid, named lamesticumin G (**100**), along with methyl lansiolate (**38**), lansiolic acid (**39**), lansionic acid (**70**), and 3β-hydroxyonocera-8(26), 14-dien-21-one (**90**), were separated from the fruit peels EtOAc fraction. They were assessed for α-glucosidase inhibition towards rat intestinal α-glucosidases (sucrase and maltase). Lamesticumin G (**100**) inhibited α-glucosidase (IC_50_ 2.27 mM), compared to acarbose (IC_50_ 0.0021 mM), while **38**, **39**, **70**, and **90** had no inhibitory potential towards maltase enzyme in the colorimetric assay [66].

### 8.8. Anti-Aging Activity

Aging is a process distinguished by the accumulation of the degenerative damages, ultimately leading to the death of an organism [124]. It is the highest risk factor for various age-linked disorders, such as diabetes, neurodegenerative disease, cancer, and stroke [125]. A wealth of research aims to develop therapies that delay age-related disorders in human. The 96% EtOH and EtOAc extracts from fruit peels (FP) and flesh (FF) of *L. domesticum* were assessed for the elastase and collagenase inhibitory activity. FP-EtOH and FP-EtOAc extracts exhibited the most potent elastase and collagenase inhibitory activity. Nevertheless, FF-EtOH extract possessed the highest tyrosinase inhibitory capacity. Therefore, the fruit flesh and peel extracts of *L. domesticum* could be a cosmetic active ingredient because of their anti-tyrosinase and anti-aging capacities [121].

### 8.9. Analgesic and Anti-Inflammatory Activities

Inflammation occurs in response to processes such as cell death, tissue injury, ischemia, cancer, and degeneration, leading to the synthesis and secretion of numerous inflammatory mediators [126]. Pain is a public health problem with considerable socioeconomic effects [127]. Its treatment needs analgesics including, anti-inflammatory agents that exhibit analgesic potential at maximum doses [128]. In this respect, the inhibition of NO (nitric oxide) and PGE2 (prostaglandin E2) production has been established as a potential therapy for different inflammatory disorders [126,129]. Several available analgesics and anti-inflammatory drugs possess adverse effects [130]. Accordingly, medicinal plants can represent a significant source of natural and safer new drugs for treating pain and inflammation [129].

Purification of the leaves EtOAc-soluble fraction yielded two new metabolites: 17(20)*E*-dyscusin B (**101**) and 17(20)*Z*-dyscusin B (**102**), along with **64**, **103**, and **104**. Compounds **101** and **102** were a pair of Δ^17(20)^ geometric isomers of pregnane steroids as established by NMR, MS, and IR analyses (Figure 10). Compounds **101** and **102** showed the significant NO inhibition in LPS-stimulated RAW264.7 cells (IC_50_s 9.13 and 14.03 µM, respectively) compared with L-NMMA (IC_50_ 0.18 µM), whereas **64**, **103**, and **104** were inactive (IC_50_ > 25 µM) in the colorimetric MTS assay [86]. Apridamayanti et al., reported that stem bark infusion had analgesic capacity with dose of 65, 130, and 195 mg/kg BW towards 0.6% acetic acid-induced writhing in mice, with writhing protection at the percentage of 57.52, 42.48, and 24.51%, respectively [120].

### 8.10. Antibacterial Activity

Antibiotics represent one of the most substantial interventions in human medicine [131]. However, the world has witnessed an alarming rise in the failure of many antibiotics to treat bacterial infections due to the generation of antibiotic-resistant and antibiotic-tolerant persister cells and biofilms [132,133]. Natural products from various sources may contribute to the discovery of novel therapeutics for multi-drug resistant bacterial infections.

Marfori et al., isolated lansioside D (**105**) from acetone fraction of EtOAc extract of the fruit peel that exhibited pronounced antibacterial activity against *S. aureus* and *B. subtilis* with MICs 31.25 and 15.62 μg/mL, respectively. It was moderately active versus *E. coli* (MIC 250 μg/mL) and inactive against *Candida lipolytica, Saccharomyces cerevisiae, Cladosporium herbarum,* and *Aspergillus niger* [92].

Ragasa et al., isolated five new onoceranoid triterpenes: lansioside C (**37**), lansiolic acid (**39**), *α*,*γ*-onoceradienedione (**52**), lansionic acid (**70**), and 3*β*-hydroxyonocera-8(26),14-dien-21-one (**90**) from the peels CH_2_Cl_2_ extract and germacrene D (**1**) and lansiolic acid (**39**) from the seeds. All compounds exhibited antibacterial potential versus *P. aeruginosa* (IZDs ranging from 11.0–13.0 mm) compared to chloramphenicol (IZD 8.0 mm CZ), where **52** had the highest potential with IZD 13.0 mm. Compounds **70** and **37** had low and moderate activities against *B. subtilis*, respectively, while **39** and **37** had low effectiveness towards *S. aureus* using the agar well method (Conc. 60 µg/well). On the other hand, they had moderate potential towards *A. niger* and *C. albicans* and low effect against *T. mentagrophytes* [58].

**Figure 11:**
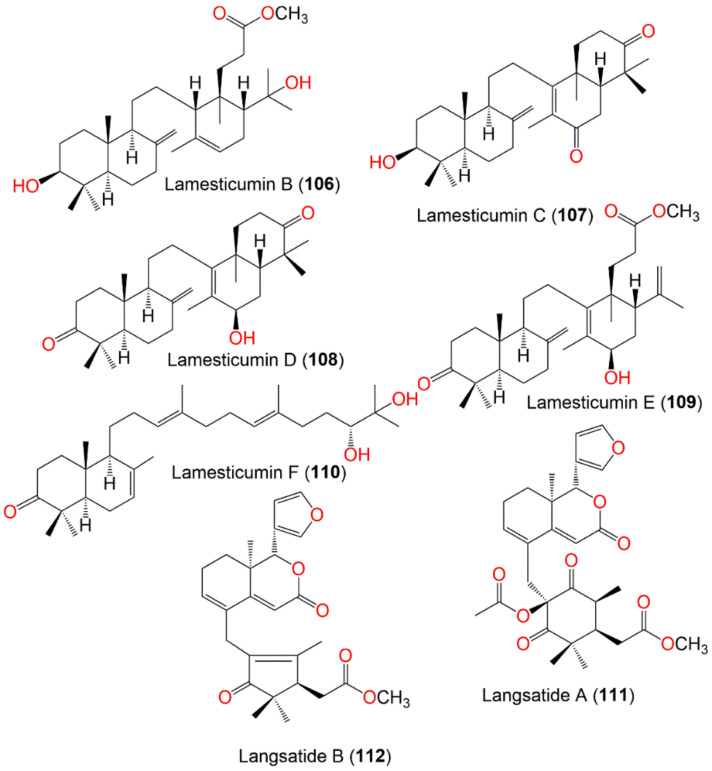
Structures of compounds **106**–**112** from *Lansium domesticum*.

Dong et al., reported the purification of structurally rare onoceranoid-triterpenoids; lamesticumins A–F (**85** and **106–110**) from the EtOAc fraction of twigs using SiO_2_ and RP_18_ CC that were elucidated based on spectroscopic analysis and the C-21 absolute configuration of **110** was assigned by Snatzke’s method (Figure 11). Compounds **85**, **106**, and **107** possessed notable activity versus *S. aureus, S. epidermidis, M. luteus, B. subtilis, M. pyogenes*, and *B. cereus* with MIC values ranging from 3.12–12.6 µg/mL, compared to magnolol (MIC 12.5–25.0 µg/mL) in the microdilution assay [87].

In 2018, Mayanti et al., isolated and characterized **48**, **51**, and **53** from the barks and leaves of *L. domesticum*. Compounds **51** and **48** possessed antibacterial effectiveness towards *E. coli* with IZDs 7.5 and 8.0 and 7.5 and 10.0 mm at concentration 500 and 1000 ppm, respectively, in comparison to vancomycin, chloramphenicol, and sulphonamide (IZDs 17.5, 18.5, and 9 mm, respectively), whereas they were inactive versus *B. cereus* in the disc diffusion assay. Compound **53** exhibited activity (Conc. 10,000, 5000, and 1000 ppm) towards *E. coli* with IZDs 3.67, 3.17, and 2.32 mm, respectively; however, they showed no activity towards *E. feacali* [77].

Furthermore, 2 new tetranortriterpenoids, langsatides A (**111**) and B (**112**), together with **40**–**43** and **45** were obtained from the seeds EtOAc fraction (Figure 11). Compounds **111** and **112** were elucidated based on spectroscopic analyses and [α]_D_ values. They showed no antibacterial potential towards *S. aureus*, *E. faecalis*, *E. Faecium*, and *Acinetobacter baumanni* (IC_50_ > 100 μM) [71].

The *L. domesticum* seeds extract showed antibacterial potential towards *S. aureus* and *E. coli* at concentration 1250 and 1000 µg/mL, respectively, in the dilution broth technique using Mueller-Hinton broth [28].

### 8.11. 5α-Reductase Inhibitory Activity

5α-Reductase is the key enzyme responsible for the biosynthesis of dihydrotestosterone (DHT) [134]. Its inhibitors are useful treatments for DHT-dependent disorders, including androgenic alopecia and hair growth, benign prostatic hyperplasia, and acne [135]. Lansiosides A–C isolated from dried peel possessed 5α-reductase inhibitory potential. They were effective in controlling male hormone-type baldness, acne, and prostate hypertrophy [58,64].

### 8.12. Wound Healing Activity

Fruits’ phenolic compounds are known to exhibit wound healing potential and accelerate tissue regeneration through their antioxidant, anti-inflammatory, and antimicrobial capacities, as well as stimulation of angiogenic activities needed for wound re-epithelialization and granulation tissue formation [136,137]. Therefore, they may be favorable ingredients in nutraceutical preparations, functional foods, or cosmeceuticals [138]. *L. domesticum* had high phenolic contents.

It was reported that AgNPs have a wound healing potential for normal and burn-related wounds because of their antifungal and antibacterial activities. Additionally, Shankar et al., reported that the incorporation of the *L. domesticum* peel extract AgNPs (0.1% *w*/*w*) in Pluronic F127 gels as a delivery system enhanced the wound healing potential. These AgNPs increased wound closure time, hydroxyproline and collagen content, and wound tensile strength (33.41 N/cm^2^) without any inflammation. Finally, the enhanced biocompatibility and wound healing activity of *L. domesticum* AgNPs were attributed to its triterpenoids [139].

Metal oxide nanoparticles (NPs) have gained remarkable attention in the biomedical field [140]. *L. domesticum* peels triterpenoids along with amino-sugars (N-acetyl-D-glucosamine) have a strong stabilizing and reducing potential that can reduce the metal ions to nanoparticles by acting as capping agents [141]. Fruit peel extract of *L. domesticum* was used as a combined reducing and capping agent to develop eco-friendly gold (Au), silver (Ag), and gold-silver (Au-Ag) nanoparticles that were characterized by various physicochemical techniques. AgNPs inhibited the *S. aureus* and *E. coli* growth (MICs 16 and 8 μg/mL and MBCs 32 and 16 mg/mL, respectively), while Au-Ag-NPs had MICs 16 μg/mL for both *S. aureus* and *E. coli*. However, AuNPs did not display any antibacterial potential [142]. Further, the cytotoxicity and cellular activity of C2C12 cells in the presence of these NPs were assessed using MTT and Almar Blue assay, respectively. AgNPs showed decreased cellular activity (Conc. > 40 μg/mL), however, AuNPs (Conc. > 50 μg/mL) exhibited no difference in cellular activity. It is noteworthy that Au-Ag-NPs did not possess cytotoxic potential compared to AgNPs, revealing that the AuNPs content in Au-Ag-NPs prohibited the AgNPs-induced cellular damage and increased the cell viability [142].

Rahma et al., synthesized AgNPs using langsat leaf (LL) extract as the bio-reductor that were characterized by UV-Vis spectrophotometer. They significantly inhibited the *E. coli* and *S. aureus* growth (Conc. 6.25 and 12.5%, respectively) in the broth dilution method. Additionally, they displayed bactericidal potential towards *E. coli* (MBC 25%) but did not have bactericidal activity towards *S. aureus* [143].

Skin aging is a physiological process that can be induced by extrinsic and intrinsic factors [144,145]. Intrinsic aging takes place within tissue through the reduction in dermal cells, fibroblasts, and collagen production, while extrinsic aging can be produced by environmental factors, especially solar UV radiation, which leads to skin damage through the ROS (reactive oxygen species) generation [146,147]. The use of antioxidants can prevent aging [148]. Increase free radicals in the body will accelerate the production of elastase and collagenase enzymes, leading to an increase in the degradation of collagen which is the major component of connective tissue on the skin [149]. Based on the strong antioxidant activity of *L. domesticum* fruit peel, it was formulated in topical semisolid pharmaceutical preparations such as gel and cream with the EtOH extract of strawberry fruit and pomelo peel as anti-aging formula [150]. All formulas showed anti-aging potential through radical DPPH scavenging, anti-collagenase, anti-tyrosinase, and anti-elastase activities [150].

The peel ethanol extract was formulated into anti-mosquito lotion using cetyl alcohol (Conc. 3, 5, and 7%). The lotion formula (5% cetyl alcohol) was prepared in 3 lotion formulas (conc. 10, 20, and 35%). These formulas did not exhibit any edema and erythema when it was applied for 3 days on rabbits. Additionally, peel extract lotion (Conc. 20 and 35%) was effective as a mosquito repellent [151].

A combination of *L. domesticum* fruit extract and Hibiscus (Hibiscus rosa-sinensis) flower extract (LHE) caused 49.37% tyrosinase inhibition, which revealed that LHE had effectiveness as a lightening agent in cosmetics preparations [27]. After applying for 4 weeks in human skin, LHE contained lotion base significantly increased skin moisture content and reduced its melanin index in the efficacy test [27].

## 9. Safety of *L. domesticum*

The dermatological safety assessment of *L. domesticum* fruits EtOH extract was carried out clinically using ROPT (Repeated Opened Patch Test) and SCPT (Single Closed Patch Test) in >50 selected healthy volunteers. A lotion base containing 50 mg of extract was applied onto the chorioallantoic membrane and left for 20 s in contact and afterward any appearance of hemorrhage, hyperemia, and opacity on the membrane was reordered using HET-CAM (Hen’s Egg Testing of Chorioallantoic Membrane) method. ROPT revealed that the extract did not produce any allergic skin reaction or irritation. SCPT exhibited no irritation or allergic skin reaction (Conc. 1% and 3%) in all volunteers, while 5% concentration produced irritation in 1.9% of all subjects [152,153,154]. Further, *L. domesticum* fruit extract and Hibiscus (Hibiscus rosa-sinensis) flower extract (LHE) lotion safety assessment by SCPT and HET-CAM indicated that LHE was safe for human eyes and skin and could be utilized as active an ingredient in cosmetics [10,27].

## 10. Conclusions

**Figure 12:**
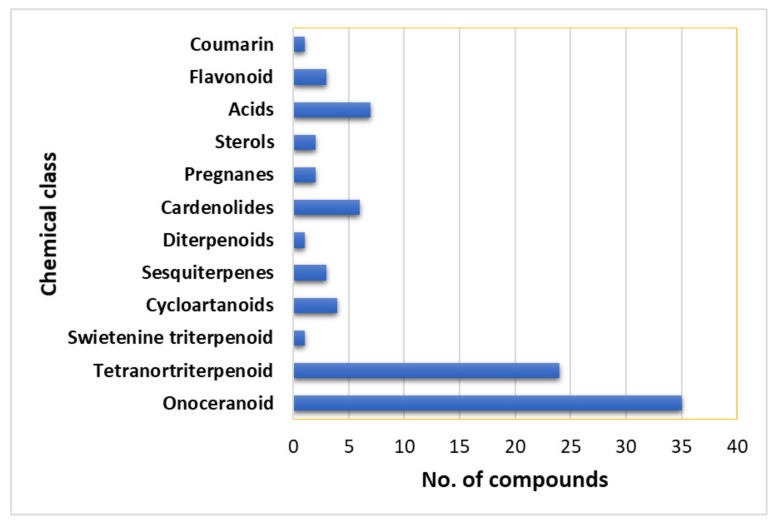
Number of metabolites reported from *Lansium domesticum* and their chemical classes.

*L. domesticum* is a commonly consumed fruit with high nutritional value, low toxicity, and long-term traditional applications for treating various diseases. The current work summarized the reported data concerning its production and season, nutritional value, phytoconstituents, enzymes, biological activities, safety, nanoparticles, and pharmaceutical formulations. It was found that yields of various plant parts MeOH extracts are varied (5.71% for peels, 6.4% for seed, and 17.94% for pulp) [53,71,155]. These percentages would vary according to the tree, source of plant material, and time of collection, as well as the extraction condition, including the technique, type of solvent, time, and temperature [53,156]. A total of 112 compounds have been reported from *L. domesticum*, including terpenes, sterols, organic acids, flavonoids, coumarin, and volatile compounds (Figure 12).

**Figure 13:**
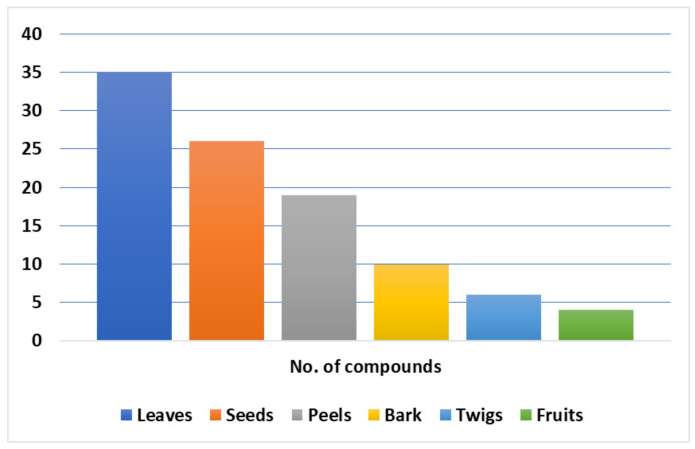
Number of metabolites reported from different part of *Lansium domesticum*.

These metabolites were isolated from the different parts of *L. domesticum* such as seeds, leaves, peels, bark, twigs, and fruits (Figure 13). Triterpenoids (64 compounds) are the major metabolites reported from *L. domesticum*, including onoceranoid (35 compounds), tetranortriterpenoid (24 compounds), cycloartane (4 compounds), and swietenine (1 compound) triterpenoids and are frequently involved in various pharmacological actions.

**Figure 14:**
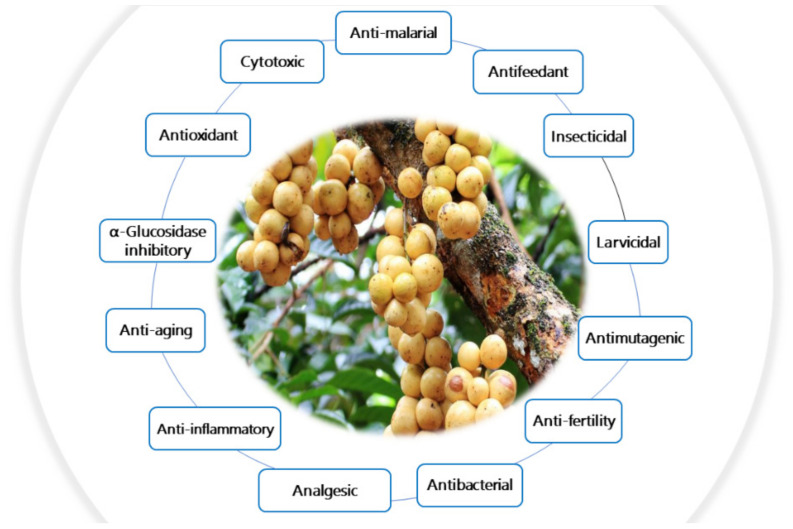
Biological activities of *L. domesticum* extracts and phytoconstituents.

Most of the reported metabolites have been evaluated for their anticancer, antifeedant, insecticidal, antidiabetic, antimalarial, antimutagenic, and antibacterial abilities (Figure 14). It is noteworthy that **94** and **95** showed potent notch inhibitory potential; therefore, they may be a candidate for anticancer or neural regenerative agents. Further, **38** and **73** had potent antimalarial effectiveness that could be further investigated for their possible use as antimalarial agents.

However, the relative study of the relationship between the structure of these metabolites and bioactivity, as well as their biosynthetic pathways is limited. The emphasis of future work should be to conduct biosynthetic pathways, possible mechanisms, and pharmacological properties of *L. domesticum* and its metabolites.

Although the phytochemical screening of *L. domesticum* revealed the existence of anthraquinones, alkaloids, and iridoids, however, none of them have been isolated as pure metabolites. Limited studies reported the synthesis of metal nanoparticles (Au- and AgNPs) using *L. domesticum* that evaluated only for their antimicrobial and wound healing potential. Therefore, future research should focus on evaluating these NPs for other bioactivities and on developing protocols for implementing the biosynthesis of NPs using other metals, metal oxides, nitrides, and carbides. Some studies reported the preparation and biological evaluation of various pharmaceutical formulations such as gel, cream, and lotion using either *L. domesticum* extracts alone or in combination with other plant extracts that proved the traditional uses of *L. domesticum* as an anti-aging, lightening, and moisturizing agent in cosmetics preparations, as well as mosquito repellent. The topical safety studies of the fruit extract revealed its safety for topical uses. Thus, future research should focus on the comprehensive utilization of *L. domesticum*, the following strategies are suggested. First, there should be an emphasis on research concerned with the single metabolite’s isolation and bioactivity evaluation rather than the crude extract. Second, metabolic pathways, structure-activity relationships, in-vivo pharmacological studies, and mechanisms of action of *L. domesticum* metabolites, particularly triterpenoids, require more attention. Third, research on the unstudied parts of *L. domesticum* that have been widely used in traditional medicine should be carried out to prove the folk use. Lastly, the toxicological evaluation of the extracts of other parts of *L. domesticum* is needed to estimate safety and reliable dosage in clinical applications. *L. domesticum* by-products (LDP) could represent wide opportunity for separating bioactive metabolites with various potential applications. Additionally, they could be a source of livestock feeds, fuel (bioethanol), or organic fertilizers [157,158]. Peels can also be utilized for the recovery of soluble dietary fibers and polyphenols [157,158]. The polyphenols recovery from the LDP can be achieved utilizing micro-, ultra-, and nano-filtration processes [159]. Therefore, proper use of these by-products will be a sustainable approach for improving health through the separation of health-promoting metabolites, as well as solving the environmental issues.

## Figures and Tables

**Table 1:** Characteristics of the major distinct forms and varieties of *L. domesticum*.

Forms/Variety	Botanical Characteristics	Ref.
Langsat	Fruits are bunched together ≈ 20 on one brown thick spike up to 20 cm length. Its fruit is oval or round ≈ 2–3 cm long and has a yellowish skin, which when peeled release a latex, showing up a translucent white flesh that is divided into segments and has 1–3 seeds. On ripping, the flesh is fairly aromatic and juicy with a sweet-acidic taste.	[11]
Duku	Fruits are bunched together ≈ 8–12, on one brown thick spike up to 20 cm length. Duku fruit is featured from langsat fruit by its larger size (3–5 cm in diameter), round shape, and much thicker skin that is comparatively free from latex. Also, it is generally more aromatic and sweeter than langsat.	[11]
Dokong (Longkong)	Fruits are occurred in bunches (25–30 fruits/bunch). Its fruit is globular with leathery, thick, and yellow skin, free of latex. The edible portion is juicy and fleshy is thin-skinned, nearly seedless, and free of latex, with uneven five-fragmented translucent white adhering aril. It has a nice aroma with a slightly sour and sweet taste.	[12,13]
Duku-langsat	It is round, brownish-yellow, and intermediate in size. It has a sweet flesh and thinner skin than that of duku.	[5]
*L. domesticum* var. *typica*	Inflorescence: rachises, young branchlets, under the surface of leaves, and calyx sparsely pubescent or sub-glabrous. Fruit: oblong-obovoid or ellipsoid, pericarp thin with little milky juice, seeds small, aril thick and smooth.	[14]
*L. domesticum* var. *pubescens* Koorders et Valeton	Inflorescence: young branchlets, rachises, calyx densely pubescent, under the surface of leaves. Fruit: sub-globose, pericarp thick with milky copious juice, thin and sour aril, large seeds.	[14]

**Table 2:** Different local names of *L. domesticum* according to the nationality [5,14,15,16].

Nationality	Name
English	Langsat, Duku
Burmese	Duku, Langsak
Filipino	Lanzone, Buahan, Lansones, Lanzon, Lansone
Indonesian	Langsat, Kokosan, Lanset Duku, Langsa, Lansot, Lasa, Lansat
Italian	Lansio, Lanzone
Malay	Langseh, Lansa, Langsep, Kokosan, Pijitan
Thai	Longkong, Duku, Langsat
Vietnamese	Bo‘N-Bon
Chinese	Lan Sa, Lan Sa Guo
Japanese	Ransa
Spanish	Arbol De Lanza, Lanzón
Portuguese	Arbol-Do-Lanza
Surinam	Duki
Malaysia	Dokong, Duku Hutan, Duku, Duku-Langsat, Langsat-Hutan, Longkong, Langsat
Korean	Lang Sat
Danish	Langsat, Langsep
French	Lansium, Langsep
Dutch	Doekoe, Langsep
Costa Rica	Duki
Cuba	Duku, Kokosan
German	Doko, Echter-Lanzebaum, Duku, Lansabaum, Langsta, Lansibaum
Honduras	Duki
Taiwan	Lan sa guo
Kenya	lengeset
Sundanese	Kokosan, Pisitan
Javanese	Langsep, langsat, celoring
Madurese	Langsep

**Table 3:** Non-medicinal and medicinal uses of *L. domesticum*.

**Forms/Variety**	**Botanical Characteristics**	**Ref.**
Fruit peels	In Java, it is dried and burned as incense in the sick people’s rooms and to repel mosquitoes. It is utilized to cure diarrhea and intestinal parasites. Fruit peels are used as an arrow poison.	[25,26]
	It is applied to the skin as a moisturizer and skin whitening cream. Borneo, it is utilized as talc powder by indigenous females of Dayak for skin protection from the sun.	[10,27]
Seeds	Pulverized seeds mixed with water are utilized as a vermifuge for children. Also, they are utilized as a febrifuge. In Peninsular Malaysia, among the Sakai the bitter seeds were crushed and utilized for curing fevers. In the Philippines, pounded seeds mixed with water are used for deworming and ulcers.	[22,23,28]
Bark	A poultice of bark used against scorpion stings. A decoction is taken for malaria and dysentery treatment in Java, Borneo, and Malaya. A tincture is useful as an anti-colic or anti-diarrhetic. In Kenya, the bark is used for spleen and fever. In Borneo, bark stew water decoction is taken by rural communities as an antifertility medicine.	[8,17,26,29,30,31]
Resin	It halts diarrhea and intestinal spasms. The resin from the bark is given for swellings, flatulence, and spasm.	[8]
Leaf	Its juice is utilized as eye drops to eleminate inflammation. A decoction of leaves and bark has been taken for curing dysentery. The Philippines used leaves for the control of mosquitoes. In Ibans in Sarawak, Malaysia leaves are used to treat fever.	[26,32,33]
Peel and flesh	It is used as facial masks, wash gels, and toners. Peel is known to be toxic to domestic animals.	[10,34]
Wood tar	It is used for blackening teeth.	[5]
Wood	It is used for tool handles, house posts, and rafters	[16]
Bark and fruit	The fruit skin‘s juice and bark are utilized as a Dyak arrow poison.	[5]
Seed and bark	A decoction of seed and bark is used for the enlargement of spleen and fever in Kenya.	[30]
Stem	The decoction of the langsat stems and bark of *Pterocarpus indica* assists treating dysentery.	[35]

**Table 5:** Biological activity of reported phytoconstituents from *Lansium domesticum*.

Compound Name	Biological Activity	Assay, Organism, or Cell Line	Biological Results		Ref.
			Compound	Positive Control	
Germacrene D (**1**)	Antimicrobial	Agar well/*Escherichia coli*	12.0 mm (CZ) *	Chloramphenicol 23.0 mm (CZ)	[58]
		Agar well/*Pseudomonas aeruginosa*	11.0 mm (CZ)	Chloramphenicol 8.0 (CZ)	[58]
		Agar well/*Candida albicans*	14.0 mm (CZ)	Chloramphenicol 10.0 mm (CZ)	[58]
		Agar well/*Aspergillus niger*	13.0 mm (CZ)	Chloramphenicol 10.0 mm (CZ)	[58]
		Agar well/*Trichophyton mentagrophytes*	13.0 mm (CZ)	Chloramphenicol 50.0 (mm (CZ)	[58]
Lansioside A (**35**)	Anti-leukotriene D_4_	leukotriene D_4_/guinea pig ileum	2.4 ppm (IC_50_)	-	[19]
Lansioside B (**36**)	Antimalarial	Microculture radioisotope/*P. falciparum* (K1, multidrugresistant strain)	>20.0 µg/mL (IC_50_)	Artemisinin 0.001–0.003 µg/mL (IC_50_)	[65]
Lansioside C (**37**)	Antimicrobial	Agar well/*Staphylococcus aureus*	19.0 mm (CZ)	Chloramphenicol 25.0 mm (CZ)	[58]
		Agar well/*Escherichia coli*	12.0 mm (CZ)	Chloramphenicol 23.0 mm (CZ)	[58]
		Agar well/*Pseudomonas aeruginosa*	12.0 mm (CZ)	Chloramphenicol 8.0 mm (CZ)	[58]
		Agar well/*Bacillus subtitis*	26.0 mm (CZ)	Chloramphenicol 20.0 mm (CZ)	[58]
		Agar well/*Candida albicans*	13.0 mm (CZ)	Chloramphenicol 10.0 mm (CZ)	[58]
		Agar well/*Aspergillus niger*	14.0 mm (CZ)	Chloramphenicol 10.0 mm (CZ)	[58]
		Agar well/*Trichophyton mentagrophytes*	20.0 mm (CZ)	Chloramphenicol 50.0 mm (CZ)	[58]
Methyl lansiolate (**38**)	Antimalarial	Microculture radioisotope/*P. falciparum* (D6, chloroquine sensitive strain)	0.65 µg/mL (IC_50_)	Artemisinin 0.0015 µg/mL (IC_50_) Chloroquine 0.0045 µg/mL (IC_50_)	[68]
		Microculture radioisotope/*P. falciparum* (W2, chloroquine resistant strain)	0.76 µg/mL (IC_50_)	Artemisinin 0.0035 µg/mL (IC_50_) Chloroquine 0.0065 µg/mL (IC_50_)	[68]
	Cytotoxicity	SRB/KB	128.0 cells % survival	-	[68]
Lansiolic acid (**39**)	Antimicrobial	Agar well/*Staphylococcus aureus*	12.0 mm (CZ)	Chloramphenicol 25.0 mm (CZ)	[58]
		Agar well/*Escherichia coli*	11.0 mm (CZ)	Chloramphenicol 23.0 mm (CZ)	[58]
		Agar well/*Pseudomonas aeruginosa*	12.0 mm (CZ)	Chloramphenicol 8.0 mm (CZ)	[58]
		Agar well/*Bacillus subtitis*	13.0 mm (CZ)	Chloramphenicol 20.0 mm (CZ)	[58]
		Agar well/*Candida albicans*	14.0 mm (CZ)	Chloramphenicol 10.0 mm (CZ)	[58]
		Agar well/*Aspergillus niger*	14.0 mm (CZ)	Chloramphenicol 10.0 mm (CZ)	[58]
		Agar well/*Trichophyton mentagrophytes*	14.0 mm (CZ)	Chloramphenicol 50.0 mm (CZ)	[58]
	Antimalarial	Microculture radioisotope/*P. falciparum* (D6, chloroquine-sensitive strain)	>10 µg/mL (IC_50_)	Artemisinin 0.0015 µg/mL (IC_50_) Chloroquine 0.0045 µg/mL (IC_50_)	[68]
		Microculture radioisotope/*P. falciparum* (W2, chloroquine- resistant strain)	>10 µg/mL (IC_50_)	Artemisinin 0.0035 µg/mL (IC_50_) Chloroquine 0.0065 µg/mL (IC_50_)	[68]
		Microculture radioisotope/*P. falciparum* (K1, multidrug-resistant strain)	>20.0 µg/mL (IC_50_)	Artemisinin 0.001–0.003 µg/mL (IC_50_)	[65]
	Cytotoxicity	SRB/KB	116.1 cells % survival	-	[68]
Dukunolide C (**42**)	Antimalarial	Microculture radioisotope/*P. falciparum* (K1, multidrug-resistant strain)	5.2 µg/mL (IC_50_)	Artemisinin 0.001–0.003 µg/mL (IC_50_)	[65]
Kokosanolide A (**47**)	Cytotoxicity	MTT/MCF-7	8.62 μg/mL (IC_50_)	-	[98]
Kokosanolide B (**48**)	Antibacterial	Disc diffusion/*E. coli*	8.0 mm (IZD)	Vancomycin 17.5 mm (IZD) Chloramphenicol 18.5 mm (IZD) Sulphonamide 9 mm (IZD)	[77]
8,14-Secogammacera-7,14-diene-3,21-dione (**51**)	Antibacterial	Disc diffusion/*E. coli*	7.5 mm (IZD)	Vancomycin 17.5 mm (IZD) Chloramphenicol 18.5 mm (IZD) Sulphonamide 9 mm (IZD)	[77]
α,γ-Onoceradienedione = 8,14-Secogammacera-7,14(27)-diene-3,21-dione (**52**)	Antimicrobial	Agar well/*Pseudomonas aeruginosa*	13.0 mm (CZ)	Chloramphenicol 8.0 mm (CZ)	[58]
		Agar well/*Candida albicans*	13.0 mm (CZ)	Chloramphenicol 10.0 mm (CZ)	[58]
		Agar well/*Aspergillus niger*	12.0 mm (CZ)	Chloramphenicol 10.0 mm (CZ)	[58]
		Agar well/*Trichophyton mentagrophytes*	13.0 mm (CZ)	Chloramphenicol 50.0 mm (CZ)	[58]
	Antimalarial	Microculture radioisotope/*P. falciparum* (D6, chloroquine sensitive strain)	1.66 µg/mL (IC_50_)	Artemisinin 0.0015 µg/mL (IC_50_) Chloroquine 0.0045 µg/mL (IC_50_)	[68]
		Microculture radioisotope/*P. falciparum* (W2, chloroquine resistant strain)	1.83 µg/mL (IC_50_)	Artemisinin 0.0035 µg/mL (IC_50_) Chloroquine 0.0065 µg/mL (IC_50_)	[68]
	Cytotoxicity	SRB/KB	131.5 cells % survival	-	[68]
		MTT/HeLa	32.39 μg/mL (IC_50_)	Doxorubicin 2.83 μg/mL (IC_50_)	[80]
		MTT/T-47D	30.69 μg/mL (IC_50_)	Doxorubicin 0.04 μg/mL (IC_50_)	[80]
		MTT/A549	13.71 μg/mL (IC_50_)	-	[80]
Domesticulide A (**58**)	Antimalarial	Microculture radioisotope/*P. falciparum* (K1, multidrug resistant strain)	>20.0 µg/mL (IC_50_)	Artemisinin 0.001–0.003 µg/mL (IC_50_)	[65]
Domesticulide B (**59**)	Antimalarial	Microculture radioisotope/*P. falciparum* (K1, multidrug resistant strain)	3.2 µg/mL (IC_50_)	Artemisinin 0.001–0.003 µg/mL (IC_50_)	[65]
Domesticulide C (**60**)	Antimalarial	Microculture radioisotope/*P. falciparum* (K1, multidrug resistant strain)	2.4 µg/mL (IC_50_)	Artemisinin 0.001–0.003 µg/mL (IC_50_)	[65]
Domesticulide D (**61**)	Antimalarial	Microculture radioisotope/*P. falciparum* (K1, multidrug resistant strain)	6.9 µg/mL (IC_50_)	Artemisinin 0.001–0.003 µg/mL (IC_50_)	[65]
Domesticulide E (**62**)	Antimalarial	Microculture radioisotope/*P. falciparum* (K1, multidrug resistant strain)	>20.0 µg/mL (IC_50_)	Artemisinin 0.001–0.003 µg/mL (IC_50_)	[65]
6-Hydroxymexicanolide (**63**)	Antimalarial	Microculture radioisotope/*P. falciparum* (K1, multidrug resistant strain)	>20.0 µg/mL (IC_50_)	Artemisinin 0.001–0.003 µg/mL (IC_50_)	[65]
6-Acetoxymexicanolide (**64**)	Antimalarial	Microculture radioisotope/*P. falciparum* (K1, multidrug resistant strain)	9.7 µg/mL (IC_50_)	Artemisinin 0.001–0.003 µg/mL (IC_50_)	[65]
Methyl angolensate (**65**)	Antimalarial	Microculture radioisotope/*P. falciparum* (K1, multidrug resistant strain)	5.9 µg/mL (IC_50_)	Artemisinin 0.001–0.003 µg/mL (IC_50_)	[65]
Methyl 6-hydroxyangolensate (**66**)	Antimalarial	Microculture radioisotope/*P. falciparum* (K1, multidrug resistant strain)	>20.0 µg/mL (IC_50_)	Artemisinin 0.001–0.003 µg/mL (IC_50_)	[65]
Methyl 6-acetoxyangolensate (**67**)	Antimalarial	Microculture radioisotope/*P. falciparum* (K1, multidrug resistant strain)	3.8 µg/mL (IC_50_)	Artemisinin 0.001–0.003 µg/mL (IC_50_)	[65]
Azadiradione (**68**)	Antimalarial	Microculture radioisotope/*P. falciparum* (K1, multidrug resistant strain)	2.9 µg/mL (IC_50_)	Artemisinin 0.001–0.003 µg/mL (IC_50_)	[65]
Onoceratriene (**69**)	Antimalarial	Microculture radioisotope/*P. falciparum* (D6, chloroquine sensitive strain)	>10 µg/mL (IC_50_)	Artemisinin 0.0015 µg/mL (IC_50_) Chloroquine 0.0045 µg/mL (IC_50_)	[68]
		Microculture radioisotope/*P. falciparum* (W2, chloroquine resistant strain)	>10 µg/mL (IC_50_)	Artemisinin 0.0035 µg/mL (IC_50_) Chloroquine 0.0065 µg/mL (IC_50_)	[68]
	Cytotoxicity	SRB/KB	108.3 cells % survival	-	[68]
Lansionic acid = 3-ketolansiolic acid (**70**)	Antimicrobial	Agar well/*Escherichia coli*	12.0 mm (CZ)	Chloramphenicol 23.0 mm (CZ)	[58]
		Agar well/*Pseudomonas aeruginosa*	12.0 mm (CZ)	Chloramphenicol 8.0 mm (CZ)	[58]
		Agar well/*Bacillus subtitis*	13.0 mm (CZ)	Chloramphenicol 20.0 mm (CZ)	[58]
		Agar well/*Candida albicans*	12.0 mm (CZ)	Chloramphenicol 10.0 mm (CZ)	[58]
		Agar well/*Aspergillus niger*	14.0 mm (CZ)	Chloramphenicol 10.0 mm (CZ)	[58]
		Agar well/*Trichophyton mentagrophytes*	15.0 mm (CZ)	Chloramphenicol 50.0 mm (CZ)	[58]
	Antimalarial	Microculture radioisotope/*P. falciparum* (D6, chloroquine-sensitive strain)	>10 µg/mL (IC_50_)	Artemisinin 0.0015 µg/mL (IC_50_) Chloroquine 0.0045 µg/mL (IC_50_)	[68]
		Microculture radioisotope/*P. falciparum* (W2, chloroquine-resistant strain)	>10 µg/mL (IC_50_)	Artemisinin 0.0035 µg/mL (IC_50_) Chloroquine 0.0065 µg/mL (IC_50_)	[68]
	Cytotoxicity	SRB/KB	129.1 cells % survival	-	[68]
Lansionic acid A = Lansiolic acid A (**71**)	Antimalarial	Microculture radioisotope/*P. falciparum* (D6, chloroquine-sensitive strain)	>10 µg/mL (IC_50_)	Artemisinin 0.0015 µg/mL (IC_50_) Chloroquine 0.0045 µg/mL (IC_50_)	[68]
		Microculture radioisotope/*P. falciparum* (W2, chloroquine resistant strain)	>10 µg/mL (IC_50_)	Artemisinin 0.0035 µg/mL (IC_50_) Chloroquine 0.0065 µg/mL (IC_50_)	[68]
	Cytotoxicity	SRB/KB	134.5 cells % survival	-	[68]
21*α*-Hydroxyonocera-8(26),14-dien-3-one = 3-keto-22-hydroxyonoceradiene (**72**)	Antimalarial	Microculture radioisotope/*P. falciparum* (D6, chloroquine-sensitive strain)	2.41 µg/mL (IC_50_)	Artemisinin 0.0015 µg/mL (IC_50_) Chloroquine 0.0045 µg/mL (IC_50_)	[68]
		Microculture radioisotope/*P. falciparum* (W2, chloroquine-resistant strain)	>10 µg/mL (IC_50_)	Artemisinin 0.0035 µg/mL (IC_50_) Chloroquine 0.0065 µg/mL (IC_50_)	[68]
	Cytotoxicity	SRB/KB	113.9 cells % survival	-	[68]
Methyl lansiolate A (**73**)	Antimalarial	Microculture radioisotope/*P. falciparum* (D6, chloroquine-sensitive strain)	0.69 µg/mL (IC_50_)	Artemisinin 0.0015 µg/mL (IC_50_) Chloroquine 0.0045 µg/mL (IC_50_)	[68]
		Microculture radioisotope/*P. falciparum* (W2, chloroquine-resistant strain)	1.02 µg/mL (IC_50_)	Artemisinin 0.0035 µg/mL (IC_50_) Chloroquine 0.0065 µg/mL (IC_50_)	[68]
	Cytotoxicity	SRB/KB	66.7 cells % survival	-	[68]
Lamesticumin A (**85**)	Antibacterial	Microdilution/*S. aureus*	6.25 µg/mL (MIC)	Magnolol 25.0 µg/mL (MIC)	[87]
		Microdilution/*S. epidermidis*	12.5 µg/mL (MIC)	Magnolol 12.5 µg/mL (MIC)	[87]
		Microdilution/*M. luteus*	6.25 µg/mL (MIC)	Magnolol 12.5 µg/mL (MIC)	[87]
		Microdilution/*B. subtilis*	3.12 µg/mL (MIC)	Magnolol 12.5 µg/mL (MIC)	[87]
		Microdilution/*M. pyogenes*	3.12 µg/mL (MIC)	Magnolol 25.0 µg/mL (MIC)	[87]
		Microdilution/*B. cereus*	3.12 µg/mL (MIC)	Magnolol 12.5 µg/mL (MIC)	[87]
	Cytotoxicity	MTT/T-47D	15.68 µg/mL (IC_50_)	Doxorubicin 0.18 µg/mL (IC_50_)	[35]
3*β*-Hydroxyonocera-8(26),14-dien-21-one (**90**)	Antimicrobial	Agar well/*Escherichia coli*	11.0 mm (CZ)	Chloramphenicol 23.0 mm (CZ)	[58]
		Agar well/*Pseudomonas aeruginosa*	12.0 mm (CZ)	Chloramphenicol 8.0 mm (CZ)	[58]
		Agar well/*Candida albicans*	14.0 mm (CZ)	Chloramphenicol 10.0 mm (CZ)	[58]
		Agar well/*Aspergillus niger*	15.0 mm (CZ)	Chloramphenicol 10.0 mm (CZ)	[58]
		Agar well/*Trichophyton mentagrophytes*	13.0 mm (CZ)	Chloramphenicol 50.0 mm (CZ)	[58]
Obebioside A (**93**)	Notch inhibitor	Luciferase/LS174T cells	1.65 μM (IC_50_)	DAPT 20 nM (IC_50_)	[90]
Honghelin (**95**)	Notch inhibitor	Luciferase/LS174T cells	0.62 μM (IC_50_)	DAPT 20 nM (IC_50_)	[90]
Obeside B (**96**)	Notch inhibitor	Luciferase/LS174T cells	0.51 μM (IC_50_)	DAPT 20 nM (IC_50_)	[90]
2-Ethyl,l,3-(2‘-menthene)propenal (**99**)	Cytotoxicity	MTT/T-47D	48.58 µg/mL (IC_50_)	Doxorubicin 0.43 µg/mL (IC_50_)	[35]
		MTT/HepG2	127.45 µg/mL (IC_50_)	Doxorubicin 1.18 µg/mL (IC_50_)	[35]
Lamesticumin G (**100**)	α-Glucosidase inhibitory	Colorimetric/Maltase	2.27 mM (IC_50_)	Acarbose 0.0021 mM (IC_50_)	[66]
17(20)*E*-dyscusin B (**101**)	NO inhibition	MTS/RAW264.7	9.13 μM (IC_50_)	L-NMMA 0.18 µM (IC_50_)	[86]
17(20)*Z*-dyscusin B (**102**)	NO inhibition	MTS/RAW264.7	14.03 μM (IC_50_)	L-NMMA 0.18 µM (IC_50_)	[86]
Lamesticumin B (**106**)	Antibacterial	Microdilution/*S. aureus*	6.25 µg/mL (MIC)	Magnolol 25.0 µg/mL (MIC)	[87]
		Microdilution/*S. epidermidis*	12.5 µg/mL (MIC)	Magnolol 12.5 µg/mL (MIC)	[87]
		Microdilution/*M. luteus*	3.12 µg/mL (MIC)	Magnolol 12.5 µg/mL (MIC)	[87]
		Microdilution/*B. subtilis*	3.12 µg/mL (MIC)	Magnolol 12.5 µg/mL (MIC)	[87]
		Microdilution/*M. pyogenes*	3.12 µg/mL (MIC)	Magnolol 25.0 µg/mL (MIC)	[87]
		Microdilution/*B. cereus*	3.12 µg/mL (MIC)	Magnolol 12.5 µg/mL (MIC)	[87]
Lamesticumin C (**107**)	Antibacterial	Microdilution/*S. aureus*	6.25 µg/mL (MIC)	Magnolol 25.0 µg/mL (MIC)	[87]
		Microdilution/*S. epidermidis*	12.5 µg/mL (MIC)	Magnolol 12.5 µg/mL (MIC)	[87]
		Microdilution/*M. luteus*	6.25 µg/mL (MIC)	Magnolol 12.5 µg/mL (MIC)	[87]
		Microdilution/*B. subtilis*	3.12 µg/mL (MIC)	Magnolol 12.5 µg/mL (MIC)	[87]
		Microdilution/*M. pyogenes*	3.12 µg/mL (MIC)	Magnolol 25.0 µg/mL (MIC)	[87]
		Microdilution/*B. cereus*	3.12 µg/mL (MIC)	Magnolol 12.5 µg/mL (MIC)	[87]
Lamesticumin D (**108**)	Antibacterial	Microdilution/*B. subtilis*	6.25 µg/mL (MIC)	Magnolol 12.5 µg/mL (MIC)	[87]
		Microdilution/*B. cereus*	3.12 µg/mL (MIC)	Magnolol 12.5 µg/mL (MIC)	[87]
Lamesticumin E (**109**)	Antibacterial	Microdilution/*B. subtilis*	12.5 µg/mL (MIC)	Magnolol 12.5 µg/mL (MIC)	[87]
		Microdilution/*M. pyogenes*	6.25 µg/mL (MIC)	Magnolol 25.0 µg/mL (MIC)	[87]
		Microdilution/*B. cereus*	3.12 µg/mL (MIC)	Magnolol 12.5 µg/mL (MIC)	[87]
Lamesticumin F (**110**)	Antibacterial	Microdilution/*B. subtilis*	12.5 µg/mL (MIC)	Magnolol 12.5 µg/mL (MIC)	[87]
		Microdilution/*B. cereus*	3.12 µg/mL (MIC)	Magnolol 12.5 µg/mL (MIC)	[87]

* CZ: clear zone.

## Data Availability

Not applicable.

## Abbreviations

A549: Lung adenocarcinoma epithelial cell line; 5-FU: 5-Fluorouracil; AgNPs: Silver nanoparticles; Au: Gold; B_16_F_10_: Human murine melanoma cells; BCB: β-Carotene bleaching assay; BHT: Butylated hydroxytoluene; C2C12: Myoblast cell line; CAT: Catalase; CC: Column chromatography; CH_2_Cl_2_: Dichloromethane; CHCl_3_: Chloroform; CYP1A2: Cytochrome P450 family 1 subfamily A member 2; CZ: Clear zone; DAPT: *N*-[*N*-(3,5-Difluorophenacetyl)-l-alanyl]-*S*-phenylglycine *t*-butyl ester; DHT: Dihydrotestosterone; DPPH: 2,2-Diphenyl-1-picrylhydrazyl; EDTA: Ethylenediaminetetraacetic acid; ERα: Estrogen receptor alpha; EtOAc: Ethyl acetate; EtOH: Ethanol; FMCA: Fluorometric microculture cytotoxicity assay; FRAP: Ferric reducing antioxidant power assay; GC-MS: Gas chromatography-mass spectrometry; GPX: Glutathione peroxidase; GSH: Glutathione; H_2_O: Water; HeLa: Human cervix carcinoma cell line; HepG2: Human hepatocarcinoma cell line; HES1: Hairy and enhancer of split 1; HETCAM: Hen’s egg-chorioallantoic membrane test; HPB-ALL: Human T cell acute lymphoblastic leukemia cells; HPLC: High performance liquid chromatograph; HRFABMS: High Resolution Fast Atom Bombardment Mass Spectrometry; HSC-F: Cynomolgus monkey normal T cells; HT-29: Human colorectal adenocarcinoma cells; IC_50_: Concentration causing 50% growth inhibition; ICP-OES: Inductively couple plasma optical emission spectrometry; IPC_50_: The sample concentrations providing 50% inhibition of lipid peroxidation; IZD: Inhibition zone diameter; KB: Human mouth epidermal carcinoma; LD_50_: The amount which causes the death of 50%; LD_90_: The amount which causes the death of 90%; L-NMMA: Nitric Oxide Synthase Inhibitor NG-Monomethyl-L-Arginine; LOX: Lipoxygenase; LS174T: Colonic adenocarcinoma cell line; LTD_4_: leukotriene D_4_; MAML: Mastermind-like protein; MC_50_: The sample concentration providing 50% metal chelating activity; MCF-7: Human breast cancer cell lines; MDA: Malondialdehyde; MeOH: Methanol; MIC: Minimum inhibitory concentrations; MMP-2: 72 kDa type IV collagenase also known as matrix metalloproteinase-2; MTS: Tetrazolium dye assays; MTT: 3-(4,5-Dimethylthiazol-2-yl)-2,5-diphenyl tetrazolium bromide; *n*-BuOH: n-Butanol; NICD: Notch intracellular domain; NMR: Nuclear magnetic resonance; NO: Nitrous oxide; NOESY: Nuclear over-Hauser effect; ODS: octadecyl silica; PG: Polygalacturonase; PGE_2_: Prostaglandin E2; PhIP; P39: 2-Amino-1-methyl-6-phenylimidazo [4,5-*b*I]pyridine; PME: Pectin methylesterase; ppm: parts per million; PTLC: preparative thin layer chromatography; RBP-J: Immunoglobulin kappa J region; ROPT: Repeated opened patch test; ROS: Reactive oxygen species; RP18: Reversed phase C_18_ silica gel; SC_50_: The sample concentrations providing 50% of scavenging; SCPT: Single Closed Patch Test; SiO_2_ CC: Silica gel column chromatography; SOD: Superoxide dismutase; SRB: Sulforhodamine B; T-47D: Human breast cancer cell line T-ALL: Human T-cell acute lymphoblastic leukemia; TLC: Thin layer chromatography; Trp-P-1; P38: 3-Amino-1,4-dimethyl-5*H*-pyrido [4,3-*b*]indole; VLC: Vacuum column chromatography; VOCs: Volatile organic compounds; WHO: World health organization.
